# The wind rose of human keratinocyte cell fate

**DOI:** 10.1007/s00018-014-1758-1

**Published:** 2014-10-18

**Authors:** Ning Wu, Xavier Gidrol

**Affiliations:** 1 Univ. Grenoble Alpes, iRTSV-BGE, 38000 Grenoble, France; 2 CEA, iRTSV-BGE, 38000 Grenoble, France; 3 INSERM, BGE, 38000 Grenoble, France; 4 Present Address: Molecular Oncology Laboratory, Institut de recherches cliniques de Montréal (IRCM), 110, avenue des Pins Ouest, Montréal, QC H2W 1R7 Canada

**Keywords:** p63, Myc, Epidermal homeostasis, Proliferation, Differentiation, Cell fate control

## Abstract

Extensive efforts have been made to understand the molecular actors that control epithelial cell fate. Although pieces of information have been obtained from single-gene function investigations, the entire picture of the molecular mechanisms involved in the regulation of epithelial homeostasis is still mysterious. Growing data indicate that gene networks rather than single “master” genes dictate cell fate. In an attempt to characterize such gene networks, we have been investigating the human keratinocyte proliferation and differentiation genes that act downstream of the transcription factor p63, a major regulator of epidermal homeostasis. We identified two networks: the cell cycle network that controls cell proliferation and the keratinocyte cell fate network. Through further analysis of the existing data on epithelial tumorigenesis and induced pluripotent stem cells, we propose a wind rose model of cell fate that is based on a balance between these two different networks that ultimately control human keratinocyte fate and epidermal homeostasis.

**Electronic supplementary material:**

The online version of this article (doi:10.1007/s00018-014-1758-1) contains supplementary material, which is available to authorized users.

## Human epidermal homeostasis

The human skin is our outermost layer that protects us against physical, chemical, and biological assaults from the external environment. The vast majority of cells in the epidermis is keratinocytes. After birth, our skin constantly renews; and the dead, cornified keratinocytes are shed off from our body and replaced by new ones originating from stem cells located in the basal layer. Human epidermal homeostasis results from exquisite control of the keratinocyte switch from the proliferative stage in the basal layer to the commitment to terminal differentiation in the suprabasal layer of the epidermis. Several studies have unraveled some of the molecular actors that participate in the regulation of epidermal homeostasis. Among them, MYC [1], NOTCH [2], WNT [3], MAPKs [4], E2F [5], RB [6], and p63 [7–10] seem particularly important. p63 is a member of the p53 protein family and is a master regulator in the control of the basal–spinous transition [9, 11]. p63 is highly expressed in the basal layer of the epidermis and participates in the maintenance of the “stemness” of keratinocytes in the interfollicular epidermis [12, 13]. Upon loss of p63, basal cells failed to divide asymmetrically, leading to the loss of stratification and differentiation [7, 13]. In an elegant study using reconstructed human epidermis, the group of Khavari showed that p63 is required for the proliferation and differentiation of developmentally mature keratinocytes via two independent mechanisms [10]. Although p63-deficient cells exhibited hypoproliferation, their inability to differentiate was not due to tissue hypoplasia. Simultaneous p63 and p53 knockdown rescued the cell proliferation defect but failed to restore differentiation in p63-knockdown cells, suggesting that defects in epidermal proliferation and differentiation are mediated via p53-dependent and -independent mechanisms, respectively. However, the underlying molecular mechanisms seem complex, and Truong et al. [10] concluded that “determining the direct downstream effectors of p63 that mediate epidermal differentiation program is of great interest for future studies”. Similarly, Elaine Fuchs indicated in an excellent review that “…identification of key genes downstream of p63 would provide important new insights into its roles in dynamic equilibrium of differentiation and proliferation” [14].

## The gene network downstream of p63 is extremely complex

Several approaches have been taken over the last few years to identify the molecular actors acting downstream of p63, notably genome-wide p63 ChIP analyzes to characterize p63 DNA targets at the genomic scale. In 2006 we undertook a ChIP-on-chip screening approach using the human keratinocyte cell line HaCaT, which predominantly expresses the ΔNp63a isoform, a truncated *N* amino terminal isoform that lacks the transactivating domain of p63 and were the first to identify 186 high-confidence p63 targets, which were validated using different biological assays [15]. We then reanalyzed these data with less stringent criteria, extended the list of targets to over 1,000, and confirmed the pivotal role of p63 in transcriptional regulation [16]. However, the genome coverage was limited in our studies. Indeed, the 12K promoter array which we used contained 12,000 “promoter regions” extending from approximately 800 bp upstream to approximately 200 bp downstream of the transcription start site, while the 12K CpG islands array contained probes with a median of approximately 300 bp, most being proximal to the transcription start site. Using a tiled, whole genome array from Affymetrix to cover a larger area of the genome, Yang et al. [17] found 5,800 target sites for p63 in the ME180 cell line. Kouwenhoven et al. [18, 19] first performed a ChIP-seq analysis of the p63 binding sites in the human genome of primary keratinocytes and found 10,895 genes that had one or more p63 binding sites within 25 kb up and downstream of the gene. A similar study recently identified 6,172 potential p63 target genes within 25 kb of p63 binding sites [19]. Together, these results suggest that gene networks acting downstream of p63 are extremely complex. This was confirmed by several transcriptome studies that compared the gene expression in human p63-depleted versus wild-type keratinocytes [10, 20]. Recently, we generated an expression profile in human keratinocytes lacking p63 and compared it with that of normal cells [21]. Despite the use of multiple biological replicates and a stringent statistical threshold (fold change ≥ 1.2 and *p* value ≤ 0.001), we characterized more than 1,000 genes that were modulated in p63-knockdown cells. Facing this complexity, we hypothesized that the regulation of epidermal homeostasis in adult humans could rely on gene networks and the dynamics of expression rather than on individual genes and absolute values of expression.

## p63 controls keratinocyte proliferation via MYC

**Fig. 1:**
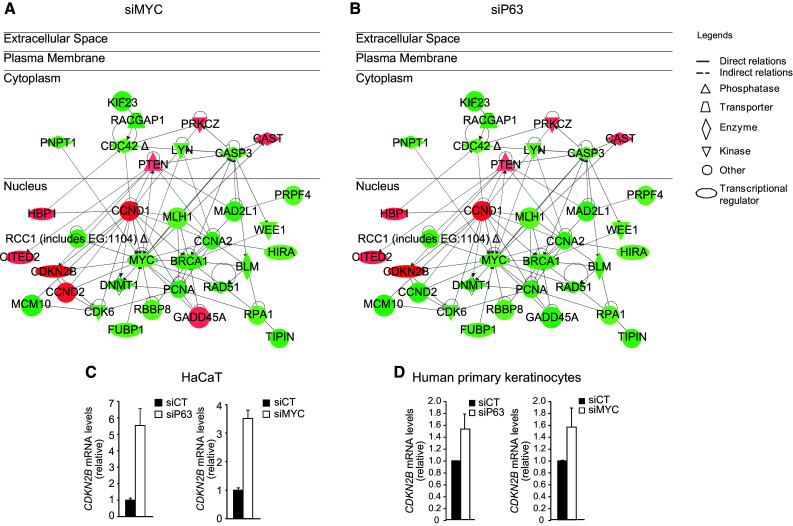
Cell cycle network that controls keratinocyte proliferation. Genetic networks regulating the cell cycle consist of common genes that are modulated in MYC- or p63-knockdown keratinocytes. The lists of genes were obtained from transcriptome profiling of HaCaT cells treated with a siRNA targeting MYC (siMYC) **a** or a siRNA targeting all isoforms of p63 (siP63) **b** after 48 h. The complete list of gene can be found in Wu el al [21]. Networks were extracted using the Ingenuity Pathway Assist software (http://www.ingenuity.com/products/ipa). Nodes (genes or proteins) in the networks are indicated by *different shapes* (biological functions) and colors (*red* indicates up-regulated, and *green* represents down-regulated). Edges are represented as *solid* or *dashed lines* to indicate direct and indirect interactions, respectively

When analyzing the expression profiles of p63-depleted keratinocytes, we soon observed that MYC was down-regulated in these cells [21]. No evidence has ever demonstrated any connection between these two genes in the regulation of epidermal homeostasis; therefore, we decided to compare the transcriptomes and phenotypic outcomes in human keratinocytes lacking either gene. Because of the existence of six different isoforms of p63, we used a siRNA targeting the conserved DNA-binding domain in all genes to achieve ablation of all p63 isoforms (siP63), and we use a siRNA targeting MYC (siMYC). The knockdown of either MYC or p63 in developmentally mature human keratinocytes resulted in impaired proliferation [21]. Because MYC does not seem to be a direct target of p63 [15, 16], the downregulation of MYC in p63-depleted keratinocytes was likely indirect. Indeed, through analysis of the MYC promoter region, we determined that p63 regulates *MYC* expression via the WNT and NOTCH signaling pathways, which, in turn, are responsible for p63-dependent regulation of MYC. Furthermore, we have characterized a cell cycle network centered on MYC that is composed of cell cycle-related genes mainly located in the nucleus and that control cell proliferation. The network is down-regulated in both genetic backgrounds, either MYC- (Fig. 1a) or p63-depleted cells (Fig. 1b). The protein p15 (CDKN2B) is a direct target of MYC [22–24], and is induced in p63- or MYC-silenced cells. It is very likely that p63 can either directly or indirectly regulate other cell cycle genes independent of MYC. Truong et al. [10] demonstrated that cell cycle arrest in p63-deficient keratinocytes was p53-dependent. Our result observed both in the HaCaT cell line (mutated p53) and in normal human primary keratinocytes (wild-type p53) demonstrated that cell cycle arrest in p63-depleted keratinocyte could also be p53-independent and MYC-dependent [21].

## The keratinocyte cell fate transcriptional network

**Fig. 2:**
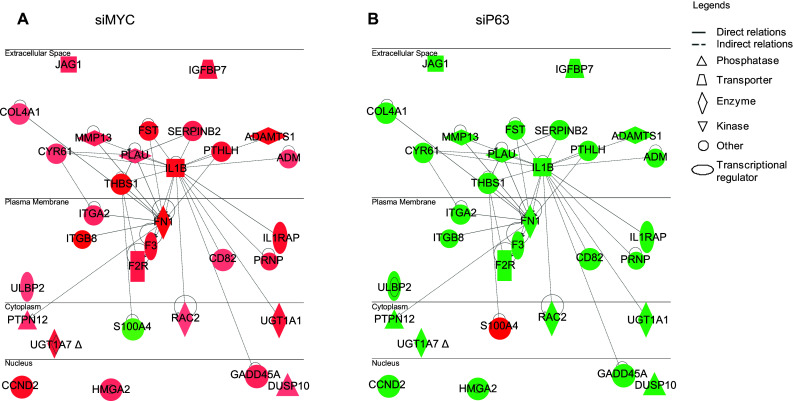
Keratinocyte cell fate network. Genetic networks involved in keratinocyte differentiation consist of common genes that are oppositely modulated in MYC- or p63-knockdown keratinocytes. The lists of genes were obtained from transcriptome profiling of HaCaT cells treated with a siRNA targeting MYC (siMYC) **a** or a siRNA targeting all isoforms of p63 (siP63) **b** after 48 h. The complete list of genes can be found in Wu et al. [21]. Networks were extracted using the Ingenuity Pathway Assist software (http://www.ingenuity.com/products/ipa). Nodes (genes or proteins) in the networks are indicated by *different shapes* (biological functions) and colors (*red* indicates up-regulated, and *green* represents down-regulated). Edges are represented as *solid* or *dashed lines* to indicate direct and indirect interactions, respectively

We observed that the ablation of p63 inhibited keratinocyte differentiation, while cells lacking MYC were still able to differentiate [21]. This confirmed that the differentiation defect was not due to tissue hypoplasia. Again, we compared the expression profiles in MYC-depleted keratinocytes with those of cells lacking p63 and identified a gene network common to p63- and MYC-knockdown keratinocytes that was oppositely regulated. This network is composed mainly of cell adhesion- and migration-related genes that are located in the cytoplasm, plasma membrane, and even secreted outside of cells (Fig. 2). Further studies demonstrated that this network plays a significant role in keratinocyte cell fate; therefore, we named it the keratinocyte cell fate (KCF) network. To summarize, the KCF network was up-regulated in keratinocytes lacking MYC (Fig. 2a) and down-regulated in p63-depleted keratinocytes (Fig. 2b). We cultured HaCaT cells in a low calcium concentration medium until confluence to induce the onset of keratinocyte differentiation. Therefore, cells began to differentiate only when they received confluence signals upon contact with other cells, and, as a consequence, it makes sense that this network is up-regulated upon keratinocyte differentiation. Strikingly, all studies on the analysis of p63 target sites in the human genome have shown enrichment in genes involved in cell adhesion: 336 genes (*p* value = 3.73E−12) [18] and 286 genes (*p* value = 1.52E−11) [19]. Furthermore, Carroll et al. [25] demonstrated that knockdown of p63 in mammary epithelial cells caused the downregulation of cell adhesion-associated genes. Lastly, numerous studies have established a clear link between cell adhesion and differentiation [26–28].

Our data suggest that upregulation of the KCF network is associated with terminal differentiation. However, whether it is the consequence or the cause of differentiation remains to be elucidated. To address this issue, the functional importance of genes such as FN1, JAG1, CYR61, and IL1B has been investigated. Interestingly, siRNA-mediated knockdown of these four genes systematically delayed the onset of terminal differentiation [21].

## The wind rose model

**Fig. 3:**
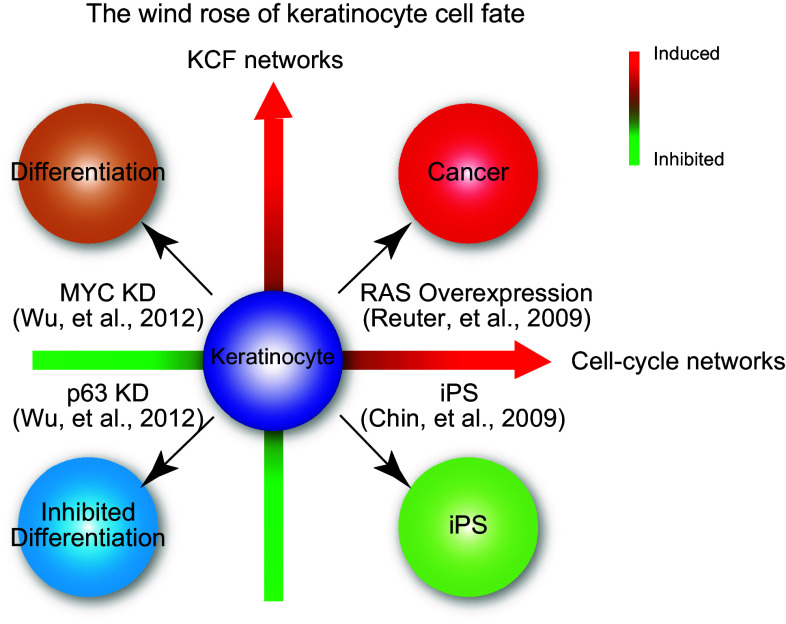
The wind rose model of human keratinocyte cells fate. The coordinated regulation of two different gene networks: the cell cycle network and the keratinocyte cell fate (KCF) network, dictates the fate of human keratinocytes and regulates epidermal homeostasis

Based on our results and other studies, we propose a “wind rose model” that dictates human KCF (Fig. 3). In this model, cell fate is determined by two gene networks: the cell cycle network, which controls cell proliferation and the KCF network. The proper balance in expression of these two networks would control the balance between cell proliferation and differentiation and eventually KCF. Different combinations of the expression levels of these two networks in response to various genetic perturbations would direct four possible phenotypic outcomes for keratinocytes: cancer, induced pluripotent stem cells, inhibited differentiation, and accelerated differentiation (Fig. 3).

In our experimental model, knockdown of p63 resulted in proliferation defects due to downregulation of the MYC-controlled cell cycle progression network (Fig. 1). This leads to proliferation defects in p63-silenced keratinocytes, further demonstrating the essential role of p63 in regulating keratinocyte proliferation. Furthermore, the lack of p63 downregulates the KCF network (Fig. 2), which is composed of genes associated with cell adhesion/migration. The decrease expression of both cell cycle and KFC networks in p63-silenced keratinocytes resulted in inhibited differentiation [21] (Fig. 3, lower-left corner of the wind rose, in blue).

If one moves clockwise through the wind rose, we next analyze the consequences of MYC silencing in human keratinocytes [21] (Fig. 3, upper-left corner of the wind rose, in orange). In MYC-depleted keratinocytes, we observed downregulation of the cell cycle network as in p63-depleted cells, whereas the KCF network was up-regulated, resulting in accelerated differentiation, unlike keratinocyte lacking p63. These results suggest that cell cycle withdrawal is necessary but not sufficient to promote differentiation.

Still moving clockwise, studies from a RAS-induced skin tumor model from Khavari’s group [29] (Fig. 3, upper-right corner of the wind rose, in red) demonstrated that a network sharing many genes with both the KCF and cell cycle networks was up-regulated upon ectopic expression of RAS. Indeed, this group reported a core tumor progression signature (CTPS) network in human keratinocytes that contained 292 nodes and was involved in carcinogenesis. This CTPS network contained several oncogene hubs, and 8 of the top 10 nodes were extracellular or cell-surface proteins related to adhesion. It is noteworthy that 4 of these 8 extracellular oncogene hubs, i.e., PLAU, CYR61, FN1, and IL1, also belong to the p63-regulated KFC network that we have characterized [21].

Finishing the wind rose tour, transcriptome data from stem cells show that 7 hubs that we identified in the KCF network, PLAU, FN1, IL1B, ADM, DUSP10, GADD45 and RAC2 [21] were significantly down-regulated in embryonic stem cells and in induced pluripotent stem (iPS) cells when compared with fibroblasts [30] (Fig. 3, lower-right corner of the wind rose, in green). Strikingly, the transcriptomics signature of human iPS shows downregulation of 6 of the 8 oncogene hubs that were reported in both the CTPS and KFC networks [30]. On the contrary, the MYC-centered cell cycle network is up-regulated in iPS cells as indeed ectopic expression of MYC, along with 3 other genes, is necessary for iPS generation [30].

In conclusion, we believe that the proposed “wind rose model” reconciles much of the existing data on the regulation of the balance between proliferation and differentiation in human skin cells and the regulation of epithelial homeostasis. Lastly, this model may enable the generation of new hypothesis and therapeutic strategies for skin diseases, including cancer.

### Electronic supplementary material

Below is the link to the electronic supplementary material.

Supplementary material 1 (DOCX 1221 kb)

Supplementary material 2 (PDF 3514 kb)
