# Supplementary material for: The wind rose of human keratinocyte cell fate
Source: Cell Mol Life Sci. 2014 Oct 18;71(24):4697–702. doi: 10.1007/s00018-014-1758-1 (PMC4233109; doi:10.1007/s00018-014-1758-1)
Supplement: Supplementary file 1 — Supplementary material 1 (DOCX 1221 kb) [file 18_2014_1758_MOESM1_ESM.docx]

Dear Johannes and Prof. Eichmann,

We thank you for your comments and positive impression concerning our manuscript. Please find below our response to each point raised by reviewers.

Sincerely,

Xavier Gidrol

Reviewer's comments:

**Reviewer #1**:

The manuscript by Wu and Gidrol presents a comparative data analysis of gene networks involved in the control of keratinocyte proliferation and differentiation. The authors mainly focused on gene expression analysis in p63 or MYC-depleted cells (HaCaT).

Despite the interesting results regarding the gene networks, the manuscript requires extensive editorial work and the addition of more specific informations to complete the presented data and to allow the comprehension from a reader of any field.

Even though the use of a cell line represents a good model for preliminary study, it is necessary to further confirm gene expression data by using primary cells, therefore using a physiological context to analyze keratinocyte differentiation (see page 5, line 48-54).

We were initially invited to write a review based on our recent “Journal of Biological Chemistry” paper (please find attached) in the “Visions and Reflections” section. To our understanding this type of article provides a forum for expert opinions on recent developments in research fields of general interest, including the opportunity of informed speculation on present and future developments. In agreement with the scope of this section we took the liberty to use some informed speculation, even though most of our findings were also confirmed in primary cells in our JBC manuscript. We describe them in detail below.

Specific comments are the following:

- Please specify the use of the terms "siP63" or "siMYC" in this manuscript as well. Is "siP63" inhibiting all p63 isoforms?

**Because of the existence of six different isoforms of p63, we used a siRNA targeting the conserved DNA binding domain in all genes to achieve ablation of all p63 isoforms**. This sentence was added in the modified ms (all modifications are in red in the new ms). As requested the terms “siP63” or “siMYC” were better specified in the text and figure legends.

- Page 5, line 31: Please consider the addition of hystological sections to explain the comparison between p63 or MYC ablation in skin. Several models have been published, most recently from Flores or Missero groups (2014).

Although several important discoveries in skin biology have been made in mice, we deliberately eliminated the mouse model and choose to focus our research and our review on human skin, as reflected in the title. Indeed, unlike other organs which are quite similar between mice and human, obviously the human skin does not resemble the mouse one. It possesses a very different structure (e.g. thinner and fewer layers in the epidermis) and striking difference in the secondary appendages, such as hair follicle. As consequence organotypic cultures and reconstructed human epidermis are probably the best models available, so the reviewer’s suggestion is interesting and relevant and we ourselves considered adding histological sections of reconstructed epidermis, but unfortunately it’s difficult to obtain such data in humans. Even though we and others reconstruct human epidermis from cells depleted in p63 (see images below), we did not find any data on human reconstructed epidermis from cells lacking MYC, as unfortunately in absence of MYC human keratinocyte proliferation is so low that epidermis cannot be reconstructed.

Eventually, we think that our message will be clearer without the addition of these histological sections.

Our own human reconstructed epidermis, after 9 days, from cell depleted in p63 (Wu et al unpublished)

Human reconstructed epidermis from Truong et al, Gene & Dev 2006, 20: 3185-3196

- Figure 1 and 2 are unclear. Please consider to enlarge both networks and increase the size of the symbol indicating the biological functions. An additional table containing gene name, Fold-Change, q-value is strongly suggested.

The figures have been modified accordingly. The tables requested by the reviewer are presented as very large Table 3 in our JBC paper (please find the paper attached). We mentioned it in legends of the revised ms and cite our JBC paper.

Besides the editorial issues, the proliferation or KCF network generated by IPA should be corroborated by supplementary experiments in primary cells.

Most of our studies have been done with a spontaneously immortalized human keratinocyte cell line HaCaT. HaCaT represents a reliable in vitro model, as most of the metabolic pathways and toxicity mechanisms remain functional (Altenburger R, Pharmaceutical Res, 1999; Bonnekoh B, Arch Dermatol Res, 1990). Furthermore, the full epidermal differentiation capacity of HaCaT cells was also demonstrated through transplantation onto nude mouse skin (Boukamp P, JCB, 1982) and reconstruction of epidermal tissue by organotypic culture *in vitro* (Maas-Szabowski N, JCS, 2003).

- Altenburger, R., and Kissel, T. (1999). The human keratinocyte cell line HaCaT: an in vitro cell culture model for keratinocyte testosterone metabolism. Pharm Res *16*, 766-771.
- Bonnekoh, B., Farkas, B., Geisel, J., and Mahrle, G. (1990). Lactate dehydrogenase release as an indicator of dithranol-induced membrane injury in cultured human keratinocytes. A time profile study. Arch Dermatol Res *282*, 325-329.
- Boukamp, P., Petrussevska, R.T., Breitkreutz, D., Hornung, J., Markham, A., and Fusenig, N.E. (1988). Normal keratinization in a spontaneously immortalized aneuploid human keratinocyte cell line. J Cell Biol *106*, 761-771.
- Maas-Szabowski, N., Starker, A., and Fusenig, N.E. (2003). Epidermal tissue regeneration and stromal interaction in HaCaT cells is initiated by TGF-alpha. J Cell Sci *116*, 2937-2948.

Nevertheless, we initially aimed at using primary cells only. But our preliminary experiments on in vitro differentiation and expression profiling in primary cells demonstrated extreme variability of responses from donor to donor. We therefore decided to focus on the HaCaT cell line to extract reliable networks. However all the major results we obtained have been confirmed in primary cells in our JBC paper (please see the attached paper).

- Influence of either MYC or p63 knockdown on keratinocyte differentiation (Fig 1H)
- Cell cycle arrest in cells treated with either siP63 or siMYC (Fig 2F & 2G)
- Knockdown of p63 down-regulates MYC (Fig 3A & 3B)
- Keratinocyte differentiation effectors (Fig 5D)

But it is clear that we are also using informed speculation in the present review.

Based on Figure 1, it seems like "cell cycle" is the most appropriate terms for the networks.

The reviewer is right. The name is more appropriate and was changed accordingly in text and figures.

What is the correlation with p53 activity in the context of p63 or MYC downregulation? Any support from in vivo analysis?

HaCaT cells are p53 mutated and primary keratinocytes were p53 wild type. We have added the following sentence in the ms, page5, to mention that : **“Our result observed both in the HaCaT cell line (mutated p53) and in normal human primary keratinocytes (wild-type p53) demonstrated that cell cycle arrest in p63-depleted keratinocyte could also be, p53-independent and MYC-dependent [21]**”.

- page 6-7. In the last paraghaph, the authors referred to RAS or iPS related network in order to define the overlap/correlation with proliferation or KCF network. It is strongly suggested to rewrite this part and explain better the connection hubs generated by the different systems.

This part of the paper has been rewritten in an effort to make it clearer. All modified parts are in red in the new ms.

**Reviewer #2**:

Wu et al. summarize in this article the molecular mechanism of proliferation and differentiation of keratinocytes focusing on the roles of p63 and MYC and propose a wind rose model based on the results. This article would be of great interest for the readers of Cellular Molecular Life Sciences. However, there are several queries for the present version of the manuscript

1. Page 4, Line 2

The authors mention an isotype, ΔNp63a. The authors should explain this isotype.

The isotype has been better explained in the modified ms. The following sentence has been added, page 4 : **“the ΔNp63a isoform, a truncated N amino terminal isoform that lacks the transactivating domain of p63”.**

2. Page 5, the last part of the first paragraph

The authors mention p15, p21, and p16 quoting Figure 1. However, these proteins do not appear in Figure 1. The readers would be confused. Either figure 1 or this part should be corrected.

As requested, we modified the text and the figure uniquely referring to p15 as “CDKN2B” (the other name for that gene). We think that it is clearer this way, even though in our JBC paper we have also shown that p21 and p16 were induced in response to either p63 or MYC depletion.

3. Page 5, the first part of the second paragraph

The authors define the KCF network as the genes up-regulated in keratinocytes lacking MYC and down-regulated in keratinocytes lacking p63 and as what is important for differentiation but not proliferation of keratinocytes. However, the authors observed that p63 inhibited keratinocyte differentiation, while cells lacking MYC were still able to differentiate. This sounds to me that the genes up-regulated by p63 but not by MYC are important for differentiation but not proliferation of keratinocytes. It means that these genes are not necessary to be up-regulated by MYC deficiency, but expression of these genes may be unchanged by existence of MYC. The authors should explain more why their definition of the KCF network is reasonable. And they should quote an appropriate reference showing that p63 inhibited keratinocyte differentiation, while cells lacking MYC were still able to differentiate.

We have shown in our JBC paper that p63 knockdown inhibits proliferation and differentiation of human keratinocytes, while MYC-depleted keratinocytes could not proliferate but were still able to differentiate (please see attached paper Fig 1). Expression profiling in both genetic backgrounds demonstrated that MYC is down regulated in cells lacking p63 (Figure 3A, 3B and 3C in JBC paper, Figure 1 in this review), thus functionally corresponding, at least partially, to a siRNA-mediated knockdown of MYC. However, these cells exhibited completely opposite differentiation outcomes (Figure 1E). To investigate the molecular mechanisms enabling keratinocyte differentiation downstream of p63, we compared the expression profiles of p63-depleted and MYC-depleted cells. As demonstrated in Figure 5A of the JBC paper, 546 genes were common to both expression profiles. It is noteworthy that there were more genes common to both profiles than specific to the p63-depleted cells. This again suggests that part of the transcriptional response to p63 ablation in human keratinocytes was also due to the down-regulation of MYC.

Among the 546 genes common to both expression profiles, we found 71 genes that were antagonistically regulated (Table S3of the JBC). We hypothesized that these antagonistically regulated genes could mechanistically explain, at least partially, the oppose differentiation outcomes between p63- and MYC-lacking keratinocytes. We used the Ingenuity knowledge base using IPA software to analyze the networks and functions associated with these 71 genes. Strikingly, a network of 41 nodes was extracted and significantly associated with a single function, cell migration/adhesion (*p*<3×10^-14^). In cells lacking *MYC,* this network was strongly up regulated (Figure 5B of the JBC paper), while in p63-depleted keratinocytes, this same network was down regulated (Figure 5C of the JBC paper).

To validate our hypothesis, we used several functional approaches. First, we searched for known phenotypes associated with these 41 genes in the Mouse Genome Informatics database and found that many of the corresponding knockout mice exhibited abnormal skin phenotypes (Table 1 of the JBC paper). To our surprise, most genes (15/19) implicated in the abnormal skin phenotypes reported in that database were also found in this network. Finally, if the genes belonging to this network promote commitment to differentiation we postulated that their expression should be down-regulated in non-differentiated and/or pluripotent cells. We data-mined the NCBI Gene Expression Omnibus database and interestingly, we found that 7 hubs in this network, *PLAU*, *FN1*, *IL1B*, *ADM*, *DUSP10*, *GADD45A*, *RAC2,* were significantly down-regulated in induced pluripotent stem cells (iPS) and are even part of the iPS transcriptomic signature (Figure 6D of the JBC paper and see image below).

The reviewer is perfectly right and we cannot eliminate that some genes up-regulated by p63, but not by MYC, could participate in the regulation of human keratinocyte differentiation. I even think that it is likely the case. Nevertheless as mentioned above, I believe we show strong evidences (Mouse phenotypes, down regulation in IPS, numerous papers showing a link between adhesion and differentiation) to demonstrate that the KFC network we have found is indeed involved in differentiation. Furthermore, as mentioned in the introduction of the present paper our hypothesis was to think in term of networks rather than individual genes in order to construct our wind rose model. Without the KFC network we could not propose the wind rose model.

This part of the paper has been rewritten accordingly in an effort to make it clearer. All modified parts are in red in the new ms.

4. Page 7, the second paragraph

The authors should mention up-regulation of the proliferation network or MYC-inducing genes in embryonic stem cells and in induced pluripotent stem cells.

This has been modified accordingly in red in the new ms. The following sentence was added, page 7. “**On the contrary the MYC-centered cell cycle network is up regulated in iPS cells as indeed ectopic expression of MYC, along with 3 other genes, is necessary for iPS generation [30]**”.
