# Supplementary material for: The wind rose of human keratinocyte cell fate
Source: Cell Mol Life Sci. 2014 Oct 18;71(24):4697–702. doi: 10.1007/s00018-014-1758-1 (PMC4233109; doi:10.1007/s00018-014-1758-1)
Supplement: Supplementary file 2 — Supplementary material 2 (PDF 3514 kb) [file 18_2014_1758_MOESM2_ESM.pdf]

p63 regulates human keratinocyte proliferation via a MYC-regulated gene network and differentiation commitment through a cell adhesion-related gene network

Ning Wu[1,2,3,#], Jérome Rollin[4], Ingrid Masse[5], Jérôme Lamartine[5] and Xavier Gidrol[1,2,3,*]

1 - CEA, IRTSV, Biologie à Grande Echelle, F-38054 Grenoble, France.

2 - INSERM, U1038, F-38054 Grenoble, France.

3 - Université Joseph Fourier, Grenoble 1, F-38000, France.

4 - CNRS, UMR6239, Laboratoire d'hématologie-hémostase, CHRU-Tours, Tours, France

5 - Université Lyon 1, CNRS, UMR5534, Centre de Génétique et de Physiologie Moléculaire et Cellulaire, 69622 Villeurbanne, France

Running title: p63 and keratinocyte cell fate

*Corresponding author. Tel 33438789796; Fax 33438785917; Email: xavier.gidrol@cea.fr

#Current address: Molecular oncology laboratory, Institut de recherches cliniques de Montréal (IRCM), 110, avenue des Pins Ouest, Montréal (Québec) H2W 1R7, Canada

Background: If the role of p63 in skin development is well known, its action in regulating human adult skin homeostasis is not completely elucidated.

Results: p63 regulates keratinocyte proliferation via a MYC-regulated gene network and differentiation through a cell adhesion-related gene network.

Conclusion: The balance between these two networks may control skin homeostasis.

Significance: This is important to understand epithelial carcinogenesis.

Although p63 and MYC are important in the control of epidermal homeostasis, the underlying molecular mechanisms governing keratinocyte proliferation or differentiation, downstream of these two genes are not completely understood. By analyzing the transcriptional changes and phenotypic consequences of the loss of either p63 or MYC in human developmentally mature keratinocytes, we have characterized the networks acting downstream of these two genes to control epidermis homeostasis. We show that p63 is required to maintain growth and to commit to differentiation by two distinct mechanisms. Knockdown of p63 led to downregulation of MYC via the Wnt/β-catenin and Notch signaling pathways and in turn reduced keratinocyte proliferation. We demonstrate that a p63-controlled keratinocyte cell fate (KCF) network is essential to induce the onset of keratinocyte differentiation. This network contains several secreted proteins involved in cell migration/adhesion, including fibronectin 1 (FN1), interleukin 1 beta (IL1B), cysteine-rich protein 61 (CYR61), and jagged-1 (JAG1), that act downstream of p63 as key effectors to trigger differentiation. Our results characterized for the first time a connection between p63 and MYC and a cell adhesion-related network that control differentiation. Furthermore, we show that the balance between the MYC-controlled cell-cycle progression network and the p63-controlled cell adhesion-related network could dictate skin cell fate.

The outer layer of human skin, the epidermis, is a self-renewing, stratified squamous epithelial tissue. In adult tissue, the maintenance of epidermal homeostasis depends on an exquisite regulation of the balance between keratinocyte proliferation and differentiation (1,2). However, the specific molecular mechanisms governing each of these processes are not completely understood.

p63, a member of the *p53* tumor suppressor gene family, is known as a key

regulator of epidermis development and keratinocyte differentiation. Striking developmental defects have been discovered during embryonic development in *p63*-knockout mice (3,4). In addition, p63 is required for the maintenance of proliferative potential in epithelial stem cells (3,5,6), epithelial lineage commitment (4), differentiation of keratinocytes (7), and epithelial cell adhesion and survival (8). Dual roles of p63 in the initiation of epithelial stratification and maintenance of stem cell proliferative potential have been established (6). As simultaneous p63 and p53 knockdown rescued cell proliferation defect of p63 knockdown alone, but failed to restore differentiation, the roles of p63 in proliferation and differentiation of developmentally mature keratinocytes appear to be distinct (7).

In vivo, as keratinocytes commit to differentiation, they detach from basal layer and migrate outward into spinous layer, accompanied with the expression of early differentiation markers K1 and K10 (9). The effects of p63 in keratinocyte differentiation have been investigated by gain and loss of function experiments. Ectopic expression of p63 induces the early markers K1, but suppresses the expression of late differentiation markers such as loricrin and filagrin (10). Loss of p63 inhibited both stratification and differentiation in skin organotypic culture (7). However little is known on key genes acting downstream of p63 to regulate the dynamic equilibrium of keratinocytes differentiation and proliferation as well as epidermal homeostasis (11).

Similar to *p63*, the *MYC* oncogene is predominantly expressed in the basal cell layers of epidermis and is absent in suprabasal layers (12,13). *MYC* plays a vital role not only in keratinocyte proliferation (14-16) but also in accelerating the differentiation of epidermal stem cells (17-20). Specifically, MYC activation leads to epidermal stem cell proliferation, and the sustained elevated expression of MYC in turn stimulates the proliferating stem cells to enter the transit amplifying compartment, thereby initiating terminal differentiation (21). Although p63 and MYC have been independently demonstrated to be key regulators in the dynamic equilibrium between proliferation and differentiation in epidermal stem cells, no connection between p63 and MYC has been reported so far. The genetic basis underlying the processes regulated by p63 and MYC in mature keratinocytes, as well as the nature of their downstream effectors, remains mostly unknown.

We hypothesized that the regulation of the equilibrium between proliferation and differentiation of keratinocytes could rely on gene networks acting downstream of p63 and MYC, rather than on a single gene. To this end, we compared phenotypic outcomes and global gene expression profiles in differentiating human keratinocytes transiently depleted of either p63 or MYC. We demonstrate that p63 regulates the proliferation and commitment to differentiation of human mature keratinocytes by two independent mechanisms. p63 is necessary for the proper expression of MYC and in turn controls keratinocyte proliferation by regulating the expression of MYC via the Wnt/β-catenin and Notch signaling pathways. Moreover, p63 regulates human keratinocyte differentiation by controlling a network of genes implicated in cell migration and adhesion.

EXPERIMENTAL PROCEDURES

Cell culture. Primary human keratinocytes were isolated from human skin biopsy and cultured in KGM2 medium (Clonetics) on flasks coated with collagen type I (Falcon Biocoat) at 37°C and 5% CO_2. This study was approved by the ethical research committee "Comité de Protection des Personnes Sud-Est II" (CODECOH Number DC-2008-262. HaCaT is a non-tumorigenic, spontaneously transformed human keratinocyte cell line (22) generously provided by N. E. Fusenig (German Cancer Research Center, Heidelberg, Germany). HaCaT cells were grown at 37°C in a humidified incubator with 5% CO_2 in

Dulbecco's modified Eagle's medium (DMEM) containing 10% fetal bovine serum, 10^5 units/liter penicillin, 50 mg/liter streptomycin, and 2 mM GlutaMax (Invitrogen). HaCaT differentiation was induced by cultivating cells seeded in plastic flasks at 10^4 cells/cm^2 as previously described (22).

Transient transfection. siRNA transfection was used INTERFERin according to the manufacturer's instructions. All transfections were performed using 10 nM of siRNA. siRNA against MYC (SI02662611), all p63 isoforms (SI00055118), FN1 (SI02664004), IL1B (SI00012166), MMP13 (SI03049277), CYR61 (SI02626421), JAG1 (SI02780134), and All Stars negative control siRNA (1027281) were obtained from QIAGEN.
Cotransfection of siRNA with plasmids was done using an Amaxa Nucleofector II (Amaxa Biosystems) under manufacturer's recommendation. Plasmids containing the ORF of cleaved Notch 1 (NICD) (Addgene plasmid 17623) (23) or Wnt-3 (Addgene plasmid 17993) (24) were obtained from Addgene.

Cell proliferation and cell death assays. 12 hours post-transfection, HaCaT cells were cultured in serum-free medium for 24 hours. After 40 hours cultured in complete medium, cell growth was performed with ViaLight Plus kit (Cambrex) according to the manufacturer's protocol. For EdU staining, HaCaT cells were pulse labeled for 24 h with 10uM EdU (Invitrogen), 24 h after transfection by siRNA. Cells were subsequently fixed on glass coverslips with paraformaldehyde immediately after the EdU-pulse labeling. The Click-it EdU reaction was performed according to the manufacturer's instructions (Invitrogen) to label and detect EdU with the Alexa Fluor azide 555 dye. EdU staining was analyzed by BD LSRII (BD bioscience). A student t test was computed for statistical analysis. We used the fluorescence-based method LIVE/DEAD Viability/Cytotoxicity Kit (Invitrogen) to evaluate cell death. HaCaT cells transfected by siRNA at 48 h after transfection were used for staining. Live cells (i.e. cells with esterase activity and intact membranes) were labeled with Calcein AM, a green-fluorescent product, and dead cells were labeled with Ethidium homodimer-1, a redfluorescent nucleic acid stain. Cell staining was visualized with an Olympus BX61 straight microscope controlled with Metamorph software (Molecular Devices, Downington, PA19335).

Microarray experiments and genetic network analysis. For transcriptome analysis, microarrays with 25,342 spotted human oligonucleotides were used (Human-25K, Réseau National des Génopoles, France) (25). RNA was isolated, amplified, labeled, and hybridized following a published protocol (25). For each condition, three independent biological replicates were obtained and the knockdown of corresponding genes was verified before RNA extraction (Figure S2). Each biological replicate was hybridized to the arrays in 4 replicates using a dye-swap strategy. Slides were scanned with an Agilent G2565AA Microarray Scanner (Agilent Technologies). Data analyses, including intensity-dependent Lowess normalization of raw data and differential analysis, were performed with GeneSpring 7.0 software (Silicon Genetics). Differentially expressed genes were identified using ANOVA (p-value corrected with Benjamini and Hoehberg False Discovery Rate) and only genes with a fold change ≥ 1.2 and $p<0.01$ were used for further analysis.
Gene pathway and genetic network analyses were performed by Ingenuity Pathway Analysis (IPA) (Ingenuity Systems) http://www.ingenuity.com. Ontologies attached to each gene were used to classify altered genes according to main biological themes. The following criteria enter into generating the networks in the Ingenuity Pathways Analysis application:
1. We have designated the molecules of interest in the Analysis Parameters before running the analysis. Molecules that a) meet

the cut-off and/or filter criteria and which b) interact with other molecules in Ingenuity's Knowledge Base are identified as focus molecules (also called Network Eligible molecules). Focus molecules serve as the "seeds", or focal points, for generating networks.

2. Networks are preferentially enriched for focus molecules with the most extensive interactions, and for which interactions are specific with the other molecules in the network (rather than molecules that are promiscuous, those that interact with a broad selection of molecules throughout Ingenuity's knowledge base).

3. Additional non-focus molecules from the dataset and from Ingenuity's knowledge base are then recruited and added to the growing networks.

4. Networks are scored for the likelihood of finding the focus molecule(s) in that given network. The higher the score, the lower the probability that you would find the focus molecules(s) you see in a given network by random chance.

5. In the current version of the application, there is a cut-off of 35 molecules per network to keep networks to a usable size.

All the microarrays data used in this study have been deposited into the NCBI Gene Expression Omnibus under accession number GSE17394.

mRNA expression analysis. RNA was extracted with an RNeasy Mini Kit (QIAGEN). For real-time quantitative PCR, 2 μg RNA was reverse-transcribed in a total volume of 20 μl with a SuperScript II RNase H reverse transcriptase system (Invitrogen) and random primers according the manufacturer's instructions. Reverse transcription reactions were diluted to 500 μl of water, and 5 μl of the diluted cDNA was used for each quantitative PCR. Quantitative PCR was carried out with a Platinum Quantitative PCR SuperMix-UDG Kit (Invitrogen) using an ABI 7500 Fast Real-Time PCR system (Applied Biosystems). All experiments were run in triplicate, and the results were normalized to 18S rRNA expression. Primer sequences are listed in Table S1.

Immunoblotting. Total cellular protein was extracted using RIPA buffer supplemented with Protease Inhibitor Cocktail (Roche Applied Science) and 1 mM sodium orthovanadate. After quantification with a BCA protein assay kit (Pierce), 20 μg of protein from each sample was run on a NuPAGE Bis-Tris gel (Invitrogen) and then transferred to a PVDF membrane. The membranes were blocked for 1 hour at room temperature in 5% nonfat dry milk/TBST, incubated overnight at 4°C with primary antibody and then 1 hour at room temperature with HRP-conjugated secondary antibody. Detection was performed with an ECL kit (Pierce). Primary antibodies used: antibodies against TP63 (4A4, sc-8431, Santa Cruz Biotechnology), MYC (N-262, sc-764, Santa Cruz Biotechnology), cytokeratin 1 (PRB-149P, Covance), cytokeratin 10 (MMS-159S, Covance), total β-catenin (6B3, 9582, Cell Signaling), cleaved Notch 1 (NICD, Val1744, 2421, Cell Signaling), and β-actin (A3854, Sigma).

Cell cycle analysis. Cell cycle phase was determined by analyzing total DNA content using flow cytometry. HaCaT cells were synchronized by culturing in serum-free DMEM medium 24 hours before transfection. Cells were transfected using INTERFERin with 10 nM of siRNA. 48 hours post-transfection, cells were collected, fixed, and stained with a Cell Cycle Phase Determination Kit (Cayman chemical). Flow cytometry analysis was performed in a MoFlo cell sorter (DakoCytomation).

Luciferase reporter assay. HaCaT cells were cotransfected with plasmid and siRNA in 24-well plates using Amaxa Nucleofector II (Amaxa Biosystems) according to the manufacturer's recommendations. The human MYC promoter cloned into a luciferase plasmid was described previously (26). A TK-*Renilla* reporter was used as an internal normalization control. The ratio of

firefly luciferase plasmid to *Renilla* luciferase was 10:1 in each nucleofection (2 µg: 200 ng). At 48 hours post-transfection, luciferase activity was measured using a Dual-Luciferase Reporter Assay System (Promega). The luciferase reporter constructions were obtained as follows: pDel-1 ~ pDel-4 reporter (Addgene plasmid 16601, 16602, 16603, 16604) (26), TCF-4/LEF reporter plasmid from SuperArray Bioscience, YY-1 reporter plasmid from Panomics, and JUN and FOS reporter plasmid from H. van Dam (Leiden University, Netherlands).

RESULTS

siRNA-mediated depletion of either p63 or MYC decreased mature keratinocyte proliferation, but only the loss of p63 inhibited differentiation. To investigate the specific roles of MYC and p63 in human keratinocyte differentiation, we knocked down each gene in HaCaT cells using specific small interfering RNA (siRNA) and analyzed the phenotypic consequences. Because of the existence of six different isoforms of p63, we used a siRNA targeting the conserved DNA binding domain in all genes to achieve ablation of all p63 isoforms. We first verified that the expression of either MYC or p63 in siRNA-transfected keratinocytes was specifically knocked down at both the transcript (Figure 1A) and protein levels (Figure 1B) 48 hours post-transfection. Compared to cells treated with control siRNA, we observed a 70% and 90% downregulation of *MYC* and *p63* expression, respectively. It is noteworthy that a significant loss of both genes was still observable after 10 days of culture (Figure S1). Two days after siRNA transfection, we observed a reduced ATP content (Figure 1C), while both EdU (Figure 1D) and Ki67 staining (Figure 1E) significantly decreased, both in p63- or MYC-knockdown HaCaT cells. Together these data demonstrate a defect in keratinocyte proliferation upon ablation of either p63 or MYC. We next monitored the capacity of these cells to differentiate *in vitro* during 10 days of culture in the appropriate medium. As we focused on the commitment to differentiation, we chose to monitor the expression of two early differentiation markers, keratin 1 (K1) and keratin 10 (K10), rather than later ones, such as involucrin or filagrin. Indeed, K1 and K10 are markers of the basal-spinous layers transition in epidermis. Reduction of p63 levels in keratinocytes significantly inhibited the expression of K1 and K10 at the transcript level (Figure 1F). In contrast, cells lacking MYC still expressed high levels of both markers, suggesting that these cells were still able to differentiate and did so even faster than the control (Figure 1F). On the protein level, K1 was continuously induced except in cells lacking p63, which exhibited upregulation of K1 until day 8, followed by a weak decrease in expression (Figure 1G). Expression of the K10 protein started late in all conditions but became significant in MYC-depleted cells after 8 days of culture (Figure 1G). In addition, we observed that loss of p63 in cultures of primary human keratinocytes (PHK) inhibited expression of both *K1* and *K10* compared to control or MYC-depleted cells (Figure 1H). These results demonstrate that the ablation of either MYC or p63 significantly reduced human mature keratinocyte proliferation, while only the siRNA-mediated loss of p63 inhibited differentiation.

Knockdown of either MYC or p63 leads to cell cycle arrest. To determine the potential origin of the proliferation defect in keratinocytes lacking either p63 or MYC, we analyzed cell death and the cell cycle. Knockdown of either p63 or MYC triggered a significant arrest in G0/G1 (Figure 2A), without affecting cell death (Figure 2B). To identify the molecular mechanisms controlling this cell cycle arrest, we analyzed the gene expression profiles of keratinocytes depleted of either MYC or p63 after transfection (Table S2). For these experiments we have particularly insisted on robustness of the data. We performed three independent siRNA transfections, three independent RNA extractions (Figure S2),

along with several technical replicates, to generated three independent expression profiles that we then averaged for either myc- or p63-depleted keratinocytes. This transcriptome analysis showed that a small network of genes strongly associated with cell cycle regulation ($p<6.2x10^{-35}$) was significantly downregulated in keratinocytes lacking either MYC or p63 (Figure S3A and Figure 2C). The majority of genes in this network were similarly regulated in both p63-depleted and MYC-depleted cells, except *CSK2*, *GADD45A*, and *CCND2* (Figure S3A). Analysis by qRT-PCR of the major cell cycle inhibitors confirmed that *p15* and *p21* were both significantly upregulated in response to the knockdown of either MYC or p63 in HaCaT cells (Figure 2D, E). Interestingly the same trend was observed in primary keratinocytes (Figure 2F, G). We also observed that MYC, a major hub (highly connected node) in this cell cycle-controlling network, was downregulated in both HaCaT and primary keratinocyte cells lacking p63 (Figure 2C, D and F). This result suggests that p63 is necessary for the proper expression of MYC in human keratinocytes.

p63 is necessary for the proper expression of MYC. We first confirmed the downregulation of MYC expression in cells lacking p63 at the transcript level (Figure 3A) and at the protein level (Figure 3B), both in HaCaT cells and in primary human keratinocyte (PHK) cultures from two different donors. Data mining in the Gene Expression Omnibus (GEO) confirmed that other groups have measured downregulation of *MYC* in p63-deficient epithelial cell lines (Figure S4) (27). Using specific siRNA targeting either ΔNp63 or TAp63, we were able to demonstrate that only the knockdown of ΔNp63 isoforms triggered downregulation of *MYC* expression (Figure 3C). This was consistent with the fact that ΔNp63 is the isoform predominantly expressed in adult keratinocytes. Conversely overexpression of either ΔNp63 or TAp63 in HaCaT cells had no effect on *MYC* expression (Figure S5). Together these results demonstrated that p63 is necessary for the proper expression of *MYC*.

We next investigated the molecular mechanisms underlying the p63 knockdown-triggered downregulation of *MYC* expression. Using luciferase reporter constructs fused to truncated *MYC* promoter regions (Figure 3D), we characterized the sequence upstream of *MYC* and identified a putative p63-controlled region. As demonstrated in Figure 3E, this region extended from -349 to -607 bp upstream of the transcription start site. A close-up view of this region revealed the absence of the p63 consensus binding site, in agreement with our previous published results, which showed that *MYC* was not a direct target of p63 in human keratinocytes (28,29). These results suggested that the expression of *MYC* is under the indirect control of p63. The region upstream of the *MYC* gene also contains several other binding sites for transcription factors (TFs), including TCF-4 (TF responding to the Wnt/β-catenin signaling pathway), YY-1 (TF responding to the Notch signaling pathway), c-FOS, and c-JUN (AP1 TFs) (Figure 3F). Because these TF are known to play important roles in the control of keratinocyte proliferation and differentiation (30-32), we investigated whether or not they are involved in the p63 knockdown-triggered downregulation of *MYC* promoter activity. In keratinocytes lacking p63, we observed a moderate inhibition of both TCF4 or YY1-dependent luciferase activities, but no change or a slight activation in JUN and FOS-driven luciferase expression (Figure 3G). These results suggest that TCF4 and YY1, the TFs responding to the Wnt/β-catenin and Notch signaling pathways, respectively, were partially responsible for the p63-dependent downregulation of *MYC* expression.

The p63 knockdown-triggered downregulation of MYC expression is mediated by the Notch and Wnt/β-catenin signaling pathways. To determine whether the signaling cascades leading to the activation of YY1 and TCF4 are affected by

p63 levels, we scrutinized the expression profiles of keratincoytes lacking either p63 or MYC. Interestingly we found that the Wnt/β-catenin and Notch signaling cascades were indeed potently inhibited in keratinocytes lacking p63. JAG1 and DLL1, ligands of the Notch signaling pathway, were both downregulated in p63-knockdown cells (Figure 4A). In addition, several inhibitors of the Wnt/β-catenin pathway were upregulated, while several activators were downregulated, likely resulting in the inhibition of this signaling pathway (Figure 4A). At the protein level, the ablation of p63 resulted in the downregulation of both cleaved Notch1 (NICD) and total β-catenin (Figure 4B). Taken together, these results suggest that both pathways were turned down in p63-knockdown cells. Inhibition of the Notch signaling pathway in presence of p63-targeting siRNA was also indicated by the reduced expression of two reporter genes of Notch-signaling cascade, *HES1* and *HEY1* (Figure 4C). To further confirm the potential involvement of the Wnt and Notch pathways in down-regulation of MYC expression, we restored downstream signaling by transfecting HaCaT cells with either WNT3 or NICD expression vectors, together with p63-targeting siRNA or control siRNA. We observed that the over expression of either WNT3 or NCID induced the upregulation of *MYC* expression both at the transcript (Figure 4D) and protein levels (Figure 4E). This would partially restore keratinocyte proliferation. However, overexpression of those two genes was not sufficient to restore proper differentiation of keratinocytes lacking p63 (data not shown). These results suggest that the molecular role of p63 in differentiation of human mature keratinocytes is likely different from the ones that dictate their proliferation.

A cell migration/adhesion-related network acts downstream of p63 to induce the onset of keratinocyte differentiation. Our results demonstrated that *MYC* is downregulated in cells lacking p63 (Figure 2 and 3), thus functionally corresponding, at least partially, to a siRNA-mediated knockdown of *MYC*. However, these cells exhibited completely opposite differentiation outcomes (Figure 1E). To investigate the molecular mechanisms enabling keratinocyte differentiation downstream of p63, we compared the expression profiles of p63-depleted and MYC-depleted cells.

As demonstrated in Figure 5A, 546 genes were common to both expression profiles. It is noteworthy that there were more genes common to both profiles than specific to the p63-depleted cells. This again suggests that part of the transcriptional response to p63 ablation in human keratinocytes was also due to the down-regulation of MYC. The downregulated genes in keratinocytes lacking either MYC (Figure S6A) or p63 (Figure S6B) shared similar Gene Ontology (GO) terms: e.g., cell cycle, DNA replication, and DNA repair. Genes upregulated in either MYC-(Figure S6C) or p63-depleted keratinocytes (Figure S6D) also shared some GO terms, such as cellular movement or cell death.

Among the 546 genes common to both expression profiles, we found 71 genes that were antagonistically regulated (Table S3). We hypothesized that these antagonistically regulated genes could mechanistically explain, at least partially, the oppose differentiation outcomes between p63- and MYC-lacking keratinocytes. We used the Ingenuity knowledge base using IPA software to analyze the networks and functions associated with these 71 genes. Strikingly, a network of 41 nodes was extracted and significantly associated with a single function, cell migration/adhesion ($p<3\times10^{-14}$). In cells lacking p63, this network was strongly downregulated (Figure 5B), while in MYC-depleted keratinocytes, this same network was upregulated (Figure S3B). We further validated the expression of some genes in the network by qRT-PCR in HaCaT cells (Figure 5C) and in primary keratinocytes (Figure 5D). As expected, the expression of these genes was upregulated in *MYC*-depleted cells and downregulated in keratinocytes lacking p63. These results suggest that this migration/adhesion-related

gene network could contain potential effectors acting downstream of p63 to induce the onset of terminal differentiation in human keratinocytes.

To validate our hypothesis, we used several functional approaches. First, we searched for known phenotypes associated with these 41 genes network in the Mouse Genome Informatics database. There are 19 knockout mice with abnormal skin phenotypes reported in that database. Strikingly, 15 genes out of these 19 KO mice were present in this network (Table 1). These data suggest that near 80% of all known skin dysfunction-related genes belongs to the network we have characterized and functionally validate it. We also monitored in vitro differentiation of keratinocyte lacking different genes belonging to this migration/adhesion network: *FN1*, *MMP13*, *JAG1*, *IL1B* and *CYR61* (Figure 6A). Except for MMP13, we observed a strong inhibition of differentiation in cells lacking any of these genes, as demonstrated by the delayed expression of *K1* (Figure 6B) and *K10* (Figure 6C) transcripts. It is noteworthy that although the ablation of IL1B was only partial (40%), yet it significantly inhibited differentiation. Furthermore ablation of all these gene together (siCocktail) strongly inhibited keratinocyte differentiation. Finally, if the genes belonging to this network promote commitment to differentiation we postulated that their expression should be down-regulated in non-differentiated and/or pluripotent cells. We data-mined the NCBI Gene Expression Omnibus database and interestingly, we found that 7 hubs in this network, *PLAU*, *FN1*, *IL1B*, *ADM*, *DUSP10*, *GADD45A*, *RAC2,* were significantly down-regulated in induced pluripotent stem cells (iPS) and are even part of the iPS transcriptomic signature (Figure 6D) (33). Together, these results show that a p63-controlled migration/adhesion-related network plays a key role in the onset of human mature keratinocyte differentiation. As a consequence we named this network, the Keratinocyte Cell Fate (KCF) network.

DISCUSSION

Our findings confirm that sustained expression of both p63 and MYC, two major regulators of epidermal homeostasis (5-7,18,21,34,35), is required to maintain growth and differentiation of human developmentally mature keratinocytes. However, we demonstrate that their respective roles are very different, as already suggested by some authors (36). We propose a model to illustrate the distinct mechanisms of action of p63 on human developmentally mature keratinocyte proliferation and differentiation (Figure 7). P63 is required fro the proper expression of of MYC expression through the combined regulation of the Wnt/β-catenin and Notch signaling pathways, leading in turn to cell cycle regulation and cell proliferation. P63 also regulates a KCF network that contains several potential “differentiation effectors”, some located in the extracellular space. The upregulation of KCF network would promote the onset of terminal differentiation of keratinocyte (Figure 7). In this study, we show that the siRNA-mediated loss of MYC triggers downregulation of the “proliferation network” and upregulation of the KCF network, promoting human keratinocyte differentiation; in contrast, p63 knockdown downregulates both cell proliferation and mobility/adhesion-related networks, thus inhibiting differentiation. These results were observed both in HaCaT cell line (mutated p53) and in normal human primary keratinocytes (wild-type p53), suggesting that the model we propose is independent of the p53 status of skin cells.

As MYC is a transcriptional repressor of p15 and p21, two cyclin-dependant kinase inhibitors (37-39), and p63 was reported to repress p21 and p16 (40-42), it was not a surprise to observe reduced proliferation in cells lacking either gene. More surprising was the p63 knockdown-triggered downregulation of *MYC* expression that we report here for the first time. This result suggests that although MYC is not a direct target of p63 (28,29), the expression of this transcription factor is

necessary for the proper expression of MYC both in HaCaT cell line and primary human keratinocytes culture. Our results establish that MYC expression is mediated, at least in part, by the Wnt/β-catenin and Notch signaling pathways. Interestingly, both pathways are important regulators of proliferation and differentiation in epidermal stem cell maintenance and wound healing (1,43-46). The interplay between the Wnt/β-catenin and Notch pathways has been reported in epidermal homeostasis and differentiation as well (47,48). We show that both pathways act in concert downstream of p63 to control the proper expression of MYC and, in turn, regulate keratinocyte cell cycle.

While the inhibition of proliferation of cells lacking p63 was partially *MYC*-dependent, the differentiation defects appeared to be independent of *MYC*. Indeed, the siRNA-mediated loss of MYC did not impair keratinocyte commitment to terminal differentiation. The differentiation was even slightly accelerated in MYC-depleted cells. By comparing expression profiles from mature keratinocytes lacking either MYC or p63, we found 546 common genes, of which 71 were antagonistically regulated. Strikingly, from that list of 71 genes we extracted a gene network containing 41 genes, that were significantly associated ($p<3\times10^{-14}$) with a single function - cell migration/adhesion. These results are consistent with recent reports showing that p63 functions as an inhibitor of cell migration (27) and that a p53 mutant forms a complex with p63 to antagonize its cell migration-inhibitory function, leading to TGFβ-dependent metastasis (49). Similarly, it was shown that p63 regulates a cell adhesion program, including integrins, in epithelial cells (8). Although we cannot exclude the possibility that other genes among the 546 common genes, but not found in our network, might regulate early differentiation, we have clearly demonstrated that this p63-controlled cell migration/adhesion network contains several effectors acting downstream of p63 to trigger differentiation of mature keratinocytes.

Some of the differentiation effectors we have identified (such as integrins, *FN1*, *PLAU*, *JAG1*, *IL1*, and *CYR61*) are also involved in cancer progression in inducible human tissue neoplasia (50). Integrin signaling plays an important role in epidermal adhesion, growth, and differentiation (51,52). JAG1 is a ligand of the Notch signaling pathway and acts on keratinocyte differentiation (32,53). JAG1 is also transcriptional target of p63 (54). Interleukin 1 (IL1) is implicated in human epidermal keratinocyte proliferation (55) and even in the regeneration of epidermal tissue *in vitro* (56). IL1α is the active form of interleukin-1 in human epidermis. IL1β was considered non-functional in keratinocytes, but our data suggest that IL1β is necessary to induce keratinocyte differentiation.

Although, most of these “differentiation effectors” do not seem to be direct transcriptional targets of p63, the control exerted by p63 on this network was dominant. Indeed we were unable to rescue differentiation of p63-depleted keratinocytes by ectopic expression of NICD or JAG1, with the use of JAG1 or IL1β recombinant proteins, or even with an acellular matrix obtained from normal fully differentiated keratinocytes (data not shown).

We report for the first time the role of this p63-regulated cell migration/adhesion network in the commitment of developmentally mature keratinocytes to differentiation. A normal expression of this network seems to be required to trigger differentiation, while its downregulation prevents it. Furthermore, we believe that misregulation of this gene network may play a major role in tumorigenesis. Indeed, Khavari’s group recently reported a core tumor progression signature (CTPS) network in keratinocytes, which contained 282 nodes and was involved in carcinogenesis (50). This CTPS network contained several oncogene hubs, and 8 of the top 10 nodes were extracellular or cell

surface proteins. It is noteworthy that 4 out of these 8 extracellular oncogene hubs, *PLAU*, *CYR61*, *FN1*, and *IL1*, also belong to the p63-regulated cell migration/adhesion network we describe in this study. Other oncogene hubs reported in the CTPS network (50), such as *SERPINE1* and *ITGA6*, were also downregulated in human keratinocytes depleted of p63 (Table S2).

In conclusion, the siRNA-mediated loss of MYC triggers downregulation of the "proliferation network" and upregulation of the "KCF migration/adhesion-related network", promoting human keratinocyte differentiation; in contrast, p63 knockdown downregulates both cell proliferation and KCF networks, thus inhibiting differentiation. We believe that the balance between levels of expression of both cell proliferation and KCF networks, could dictate keratinocyte cell fate. Furthermore, we think that this network approach would reconcile much of the existing data on the regulation of the balance between proliferation and differentiation in skin.

ACKNOWLEDGEMENTS

NW was supported by CEA and FRM (Fondation pour la Recherche Médicale). We would like to thank Jernej Murn for its suggestions on the manuscript. The authors declare no conflict of interest.

REFERENCES

1. Fuchs, E. (2007) *Nature* **445**, 834-842
2. Fuchs, E., and Raghavan, S. (2002) *Nat Rev Genet* **3**, 199-209
3. Yang, A., Schweitzer, R., Sun, D., Kaghad, M., Walker, N., Bronson, R. T., Tabin, C., Sharpe, A., Caput, D., Crum, C., and McKeon, F. (1999) *Nature* **398**, 714-718
4. Mills, A. A., Zheng, B., Wang, X. J., Vogel, H., Roop, D. R., and Bradley, A. (1999) *Nature* **398**, 708-713
5. Senoo, M., Pinto, F., Crum, C. P., and McKeon, F. (2007) *Cell* **129**, 523-536
6. Koster, M. I., Kim, S., Mills, A. A., DeMayo, F. J., and Roop, D. R. (2004) *Genes Dev* **18**, 126-131
7. Truong, A. B., Kretz, M., Ridky, T. W., Kimmel, R., and Khavari, P. A. (2006) *Genes Dev* **20**, 3185-3197
8. Carroll, D. K., Carroll, J. S., Leong, C. O., Cheng, F., Brown, M., Mills, A. A., Brugge, J. S., and Ellisen, L. W. (2006) *Nat Cell Biol* **8**, 551-561
9. Dlugosz, A. A., and Yuspa, S. H. (1993) *J Cell Biol* **120**, 217-225
10. Ogawa, E., Okuyama, R., Egawa, T., Nagoshi, H., Obinata, M., Tagami, H., Ikawa, S., and Aiba, S. (2008) *J Biol Chem* **283**, 34241-34249
11. Fuchs, E. (2009) *Cell Stem Cell* **4**, 499-502
12. Osterland, C. K., Wilkinson, R. D., and St Louis, E. A. (1990) *Clin Exp Rheumatol* **8**, 145-150
13. Bull, J. J., Muller-Rover, S., Patel, S. V., Chronnell, C. M., McKay, I. A., and Philpott, M. P. (2001) *J Invest Dermatol* **116**, 617-622
14. Jensen, K. B., and Watt, F. M. (2006) *Proc Natl Acad Sci U S A* **103**, 11958-11963
15. Hashiro, M., Matsumoto, K., Okumura, H., Hashimoto, K., and Yoshikawa, K. (1991) *Biochem Biophys Res Commun* **174**, 287-292
16. Pietenpol, J. A., Holt, J. T., Stein, R. W., and Moses, H. L. (1990) *Proc Natl Acad Sci U S A* **87**, 3758-3762
17. Arnold, I., and Watt, F. M. (2001) *Curr Biol* **11**, 558-568
18. Gandarillas, A., and Watt, F. M. (1997) *Genes Dev* **11**, 2869-2882
19. Gebhardt, A., Frye, M., Herold, S., Benitah, S. A., Braun, K., Samans, B., Watt, F. M., Elsasser, H. P., and Eilers, M. (2006) *J Cell Biol* **172**, 139-149
20. Waikel, R. L., Kawachi, Y., Waikel, P. A., Wang, X. J., and Roop, D. R. (2001) *Nat Genet* **28**, 165-168
21. Watt, F. M., Frye, M., and Benitah, S. A. (2008) *Nat Rev Cancer* **8**, 234-242

22. Boukamp, P., Petrussevska, R. T., Breitkreutz, D., Hornung, J., Markham, A., and Fusenig, N. E. (1988) *J Cell Biol* **106**, 761-771
23. Yu, X., Alder, J. K., Chun, J. H., Friedman, A. D., Heimfeld, S., Cheng, L., and Civin, C. I. (2006) *Stem Cells* **24**, 876-888
24. Shimizu, H., Julius, M. A., Giarre, M., Zheng, Z., Brown, A. M., and Kitajewski, J. (1997) *Cell Growth Differ* **8**, 1349-1358
25. Le Brigand, K., Russell, R., Moreilhon, C., Rouillard, J. M., Jost, B., Amiot, F., Magnone, V., Bole-Feysot, C., Rostagno, P., Virolle, V., Defamie, V., Dessen, P., Williams, G., Lyons, P., Rios, G., Mari, B., Gulari, E., Kastner, P., Gidrol, X., Freeman, T. C., and Barbry, P. (2006) *Nucleic Acids Res* **34**, e87
26. He, T. C., Sparks, A. B., Rago, C., Hermeking, H., Zawel, L., da Costa, L. T., Morin, P. J., Vogelstein, B., and Kinzler, K. W. (1998) *Science* **281**, 1509-1512
27. Barbieri, C. E., Tang, L. J., Brown, K. A., and Pietenpol, J. A. (2006) *Cancer Res* **66**, 7589-7597
28. Vigano, M. A., Lamartine, J., Testoni, B., Merico, D., Alotto, D., Castagnoli, C., Robert, A., Candi, E., Melino, G., Gidrol, X., and Mantovani, R. (2006) *EMBO J* **25**, 5105-5116
29. Pozzi, S., Zambelli, F., Merico, D., Pavesi, G., Robert, A., Maltere, P., Gidrol, X., Mantovani, R., and Vigano, M. A. (2009) *PLoS ONE* **4**, e5008
30. Mehic, D., Bakiri, L., Ghannadan, M., Wagner, E. F., and Tschachler, E. (2005) *J Invest Dermatol* **124**, 212-220
31. Slavik, M. A., Allen-Hoffmann, B. L., Liu, B. Y., and Alexander, C. M. (2007) *BMC Dev Biol* **7**, 9
32. Nguyen, B. C., Lefort, K., Mandinova, A., Antonini, D., Devgan, V., Della Gatta, G., Koster, M. I., Zhang, Z., Wang, J., Tommasi di Vignano, A., Kitajewski, J., Chiorino, G., Roop, D. R., Missero, C., and Dotto, G. P. (2006) *Genes Dev* **20**, 1028-1042
33. Chin, M. H., Mason, M. J., Xie, W., Volinia, S., Singer, M., Peterson, C., Ambartsumyan, G., Aimiuwu, O., Richter, L., Zhang, J., Khvorostov, I., Ott, V., Grunstein, M., Lavon, N., Benvenisty, N., Croce, C. M., Clark, A. T., Baxter, T., Pyle, A. D., Teitell, M. A., Pelegrini, M., Plath, K., and Lowry, W. E. (2009) *Cell Stem Cell* **5**, 111-123
34. McKeon, F. (2004) *Genes Dev* **18**, 465-469
35. Dai, X., and Segre, J. A. (2004) *Curr Opin Genet Dev* **14**, 485-491
36. Truong, A. B., and Khavari, P. A. (2007) *Cell Cycle* **6**, 295-299
37. Staller, P., Peukert, K., Kiermaier, A., Seoane, J., Lukas, J., Karsunky, H., Moroy, T., Bartek, J., Massague, J., Hanel, F., and Eilers, M. (2001) *Nat Cell Biol* **3**, 392-399
38. Seoane, J., Pouponnot, C., Staller, P., Schader, M., Eilers, M., and Massague, J. (2001) *Nat Cell Biol* **3**, 400-408
39. Seoane, J., Le, H. V., and Massague, J. (2002) *Nature* **419**, 729-734
40. Su, X., Cho, M. S., Gi, Y. J., Ayanga, B. A., Sherr, C. J., and Flores, E. R. (2009) *EMBO J* **28**, 1904-1915
41. Westfall, M. D., Mays, D. J., Sniezek, J. C., and Pietenpol, J. A. (2003) *Mol Cell Biol* **23**, 2264-2276
42. Testoni, B., and Mantovani, R. (2006) *Nucleic Acids Res* **34**, 928-938
43. Blanpain, C., Lowry, W. E., Pasolli, H. A., and Fuchs, E. (2006) *Genes Dev* **20**, 3022-3035
44. Rangarajan, A., Talora, C., Okuyama, R., Nicolas, M., Mammucari, C., Oh, H., Aster, J. C., Krishna, S., Metzger, D., Chambon, P., Miele, L., Aguet, M., Radtke, F., and Dotto, G. P. (2001) *EMBO J* **20**, 3427-3436
45. Watt, F. M., Estrach, S., and Ambler, C. A. (2008) *Curr Opin Cell Biol* **20**, 171-179
46. Ito, M., Yang, Z., Andl, T., Cui, C., Kim, N., Millar, S. E., and Cotsarelis, G. (2007) *Nature* **447**, 316-320

47. Devgan, V., Mammucari, C., Millar, S. E., Brisken, C., and Dotto, G. P. (2005) *Genes Dev* **19**, 1485-1495
48. Estrach, S., Ambler, C. A., Lo Celso, C., Hozumi, K., and Watt, F. M. (2006) *Development* **133**, 4427-4438
49. Adorno, M., Cordenonsi, M., Montagner, M., Dupont, S., Wong, C., Hann, B., Solari, A., Bobisse, S., Rondina, M. B., Guzzardo, V., Parenti, A. R., Rosato, A., Bicciato, S., Balmain, A., and Piccolo, S. (2009) *Cell* **137**, 87-98
50. Reuter, J. A., Ortiz-Urda, S., Kretz, M., Garcia, J., Scholl, F. A., Pasmooij, A. M., Cassarino, D., Chang, H. Y., and Khavari, P. A. (2009) *Cancer Cell* **15**, 477-488
51. Muller, E. J., Williamson, L., Kolly, C., and Suter, M. M. (2008) *J Invest Dermatol* **128**, 501-516
52. Watt, F. M. (2002) *EMBO J* **21**, 3919-3926
53. Nickoloff, B. J., Qin, J. Z., Chaturvedi, V., Denning, M. F., Bonish, B., and Miele, L. (2002) *Cell Death Differ* **9**, 842-855
54. Sasaki, Y., Ishida, S., Morimoto, I., Yamashita, T., Kojima, T., Kihara, C., Tanaka, T., Imai, K., Nakamura, Y., and Tokino, T. (2002) *J Biol Chem* **277**, 719-724
55. Yano, S., Banno, T., Walsh, R., and Blumenberg, M. (2008) *J Cell Physiol* **214**, 1-13
56. Maas-Szabowski, N., Starker, A., and Fusenig, N. E. (2003) *J Cell Sci* **116**, 2937-2948

FIGURE LEGENDS

Figure 1. Influence of knockdown of *MYC* or *p63* on keratinocyte differentiation. (**A, B**) p63 and MYC expression was measured by qRT-PCR (A) and western blot (B) after introduction of siRNA oligonucleotide duplexes. siCT is a non-targeting siRNA control, siP63 is an siRNA duplex against all p63 isoforms, and siMYC is an siRNA targeting the MYC oncogene. mRNA expression levels were normalized to siCT (A). Samples used in panel (A) and (B) were extracted 48 hours after siRNA transfection. (**C**) Cell growth was inspected by Vialight. Error bars are s.d. of 6 biological replicates and student t test was used for statistical analysis. ***, p<0.001. (**D**) Cell proliferation was assessed by EdU incorporation measured by flow cytometry. Three independent experiments were used and a student t test was used for statistical analysis. ***, p<0.001. (**E**) Ki67 staining of HaCaT cells 48h post-transfection by siRNA. Red, Ki67; Blue, Hoechst. **(F, G)** Time course of qRT-PCR (F) and western blot (G) analysis of human cytokeratin 1 (*K1*) and cytokeratin 10 (*K10*) expression in HaCaT cells transfected with siCT, siMYC, or siP63. mRNA expression levels were normalized to day 0. β-actin was used as a loading control in the western blot. (**H**) Time course of qRT-PCR analysis of *K1* and *K10* mRNA levels in primary human keratinocytes (PHK), transfected with siCT, siMYC, or siP63. mRNA expression levels were normalized to day 0. Error bars in all qRT-PCR graphs are standard deviation (s.d.) of triplicate samples from one representative experiment.

Figure 2. Shared cell cycle arrest mechanisms in cells treated with siP63 or siMYC. (**A**) Cell cycle phase determination analysis performed by flow cytometry. HaCaT cells treated with siCT (left), siP63 (middle), or siMYC (right). PI, Propidium iodide. (**B**) Live/Dead staining of siRNA transfected HaCaT cells. Red, EthD-1; Green, calcein. (**C**) Genetic networks regulating the cell cycle was generated by Ingenuity Pathway Analysis (IPA). The lists of genes used in IPA were obtained from transcriptome analysis of HaCaT cells treated with siP63 for 48 hours**.** All cell cycle-related genes in siP63 were extracted from IPA database with a very significant p-value ($<6.2\times10^{-35}$) to generate this network (see Material and Methods). Nodes (genes or proteins) in the networks are shown by different shapes (biological functions) and colors (red indicates upregulated and green represents downregulated). Edges are represented as solid or dashed lines to indicate direct and indirect interactions, respectively. (**D, E**) qRT-PCR analysis of cyclin-dependent kinase inhibitors (CDKNs, such as *p15*, *p16*, and *p21*) and *MYC* in siP63 (D) or in siMYC (E) HaCaT keratinocytes. mRNA expression levels were normalized to siCT, and error

bars in qRT-PCR graphs are standard deviation (s.d.) of triplicate samples from one representative experiment. Student t test was used for statistical analysis. *, $p<0.05$ and **, $p<0.01$. (**F**, **G**) Identical to D and E in human primary keratinocytes lacking either p63 (F) or MYC (G).

Figure 3. Knockdown of p63 downregulates MYC. QRT-PCR (**A**) and Western blot (**B**) analysis of MYC expression when p63 was knocked down by siRNA targeting all p63 isoforms in HaCaT cells or primary human keratinocytes (PHK). (**C**) *MYC* expression in ΔNp63-, TAp63, or p63-knockdown cells. 48 hours post-transfection, *MYC* expression was measured by qRT-PCR. 18S rRNA was endogenous control to normalize the results. (**D**) Structure of MYC promoter region cloned into the luciferase reporter plasmids. The bold horizontal lines represent the promoter sequences in each reporter plasmid. (**E**) HaCaT cells were cotransfected with control siRNA (siCT) or p63 siRNA (siP63) and the *MYC* promoter luciferase reporter constructs. Luciferase activities (means ± s.d.) were measured 48 hours post-transfection. Error bars are s.d. of 5 biological replicates. Firefly luciferase luminescence was normalized to an internal control *Renilla* luciferase. (**F**) Schematic representation of MYC promoter region between -300 bp to -650 bp (which corresponds to the differences between pDel-3 and pDel-4). Known transcription factor (TF) binding sites are indicated by arrows. (**G**) HaCaT cells were cotransfected with control siRNA or all-p63 siRNA and TCF-4, YY-1, JUN, or FOS luciferase reporter plasmids (TFs marked in red in panel **G**). Detailed luminescence measurements and analysis can be found in the Materials and Methods. Error bars are s.d. of replicates. The Mann-Whitney test was used for statistical analysis in (**E**) and (**G**). *, $p<0.05$.

Figure 4. The Wnt/β-catenin and Notch pathways are responsible for the MYC downregulation in p63 knockdown keratinocytes. (**A**) List of modulators of the Notch and β-catenin pathways that were differentially expressed according to siP63 transcriptome analysis. Green arrows represent a decrease in expression and the red arrows represent an increase compared to siCT-treated cells. (**B**) Western blots of cleaved Notch intra-cellular domain (NICD), total β-catenin, and MYC in siP63 as compared to siCT. (**C**) qRT-PCR analysis of expression of *HES1* and *HEY1*, which are two transcriptional target genes of the Notch signaling pathway. The values represent the mean ± s.d. of three replicate samples from one representative experiment. (**D, E**) *MYC* expression study at mRNA level (D) and at protein level (E) after activation of the β-catenin or Notch pathway via overexpression of Wnt3 or NICD, respectively. A plasmid expressing GFP was used as a transfection control. Co-transfection of siRNA and plasmids was done by nucleofection using Amaxa (see details in Materials and Methods). Error bars in all qRT-PCR graphs represent the standard deviation (s.d.) of triplicates from one representative experiments.

Figure 5. Candidate "Keratinocyte Differentiation Effectors" identified by comparative siMYC and siP63 transcriptome analysis. (**A**) Venn diagram indicates distribution of differentially expressed genes in siMYC and siP63. 546 genes are in common in siMYC and siP63. (**B**) IPA analysis with the list of oppositely expressed genes in the common part of panel A extracted a genetic network associated with a function of cell migration with a very significant p-value ($p<3\times10^{-14}$). The genes in this network are downregulated in siP63 keratinocytes and upregulated in siMYC kertainocytes (figure S6). This network was named the Keratinocyte Cell Fate (KFC) network and contains candidate keratinocyte differentiation effectors acting downstream of p63. (**C, D**) Expression levels of genes identified in genetic networks validated by qRT-PCR in HaCaT cells (C) and primary keratinocytes (D). Student t test was used for statistical analysis. *, $p<0.05$ and **, $p<0.01$.

Figure 6. An extracellular matrix genetic network is involved in keratinocyte differentiation. (**A**) Validations of siRNA knockdown targeting *FN1*, *MMP13*, *JAG1*, *IL1B*, or *CYR61*. Cells were transfected with 10 nM siRNA for 48 hours, and then the gene expression levels were quantified by qRT-PCR. The effect of knockdown of each gene was compared with a negative control siRNA (siCT). (**B, C**) Time course analysis of *K1* (B) and *K10* (C) expression were determined by qRT-PCR 9 days post-transfection of HaCaT cells with siRNA against *FN1*, *MMP13*, *IL1B*, *JAG1, CYR61*, or siCock (cocktail of these 5 siRNA). (**D**) Expression of oncogene hubs in human induced pluripotent stem cells (iPS). hFibr, human fibroblast; e-hiPSC, early human iPS; l-hiPSC, late human iPS; hESC, human embryonic stem cells. Each spot represent an expression data from one biological sample.

Figure 7. Schematic representation of the mechanism of commitment of keratinocyte differentiation controlled by p63. p63 controls keratinocyte proliferation and differentiation independently via different genetic networks. p63 regulates the cell cycle in part by regulating MYC expression through both the Wnt/-catenin and Notch pathways. To trigger the onset of differentiation, p63 controls the KCF network composed of keratinocyte differentiation effectors. Most of these effectors, such as FN1, IL1B, JAG1, and CYR61, are located in the extracellular matrix and are implicated in cell migration.

Table 1. List of all knockout mice exhibiting abnormal skin phenotypes reported in the Mouse Genome Informatics (MGI) database. Genes which are also present in the KCF network (Fig. 5B) and are antagonistically regulated in MYC- and p63-deficient keratinocytes are shown in bold. JNK and PI3K families figure in the network and therefore no specific accession number could be given.

Figure 1

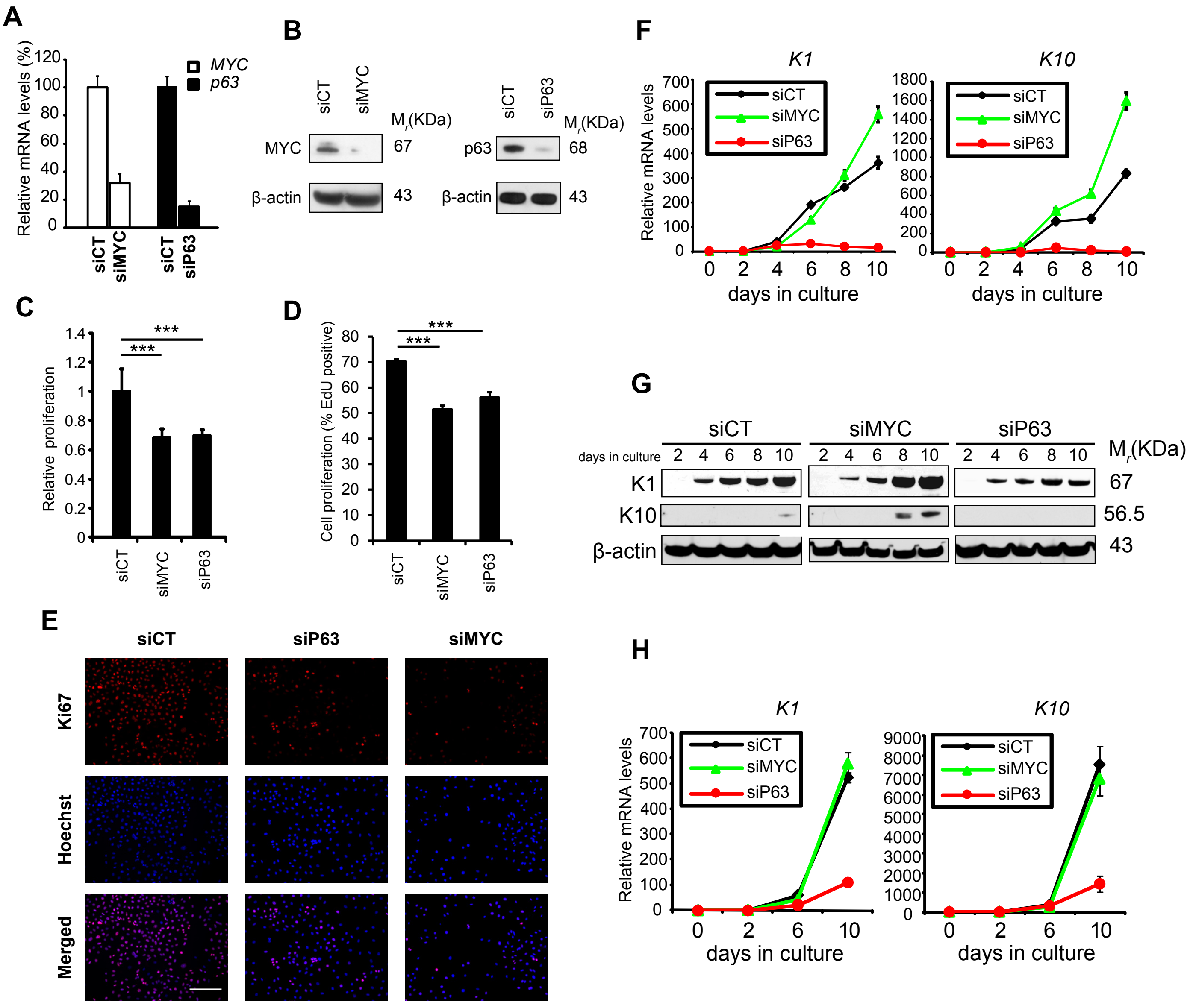
A
Relative mRNA levels (%)
MYC
p63
siCT
siMYC
siCT
siP63
B
siCT
siMYC
MYC
β-actin
M_r(KDa)
67
43
siCT
siP63
p63
β-actin
M_r(KDa)
68
43
C
Relative proliferation
siCT
siMYC
siP63
D
Cell proliferation (% EdU positive)
siCT
siMYC
siP63
E
siCT
siP63
siMYC
Ki67
Hoechst
Merged
F
K1
K10
Relative mRNA levels
siCT
siMYC
siP63
days in culture
G
siCT
siMYC
siP63
days in culture
M_r(KDa)
K1
K10
β-actin
67
56.5
43
H
K1
K10
Relative mRNA levels
siCT
siMYC
siP63
days in culture

Figure 2

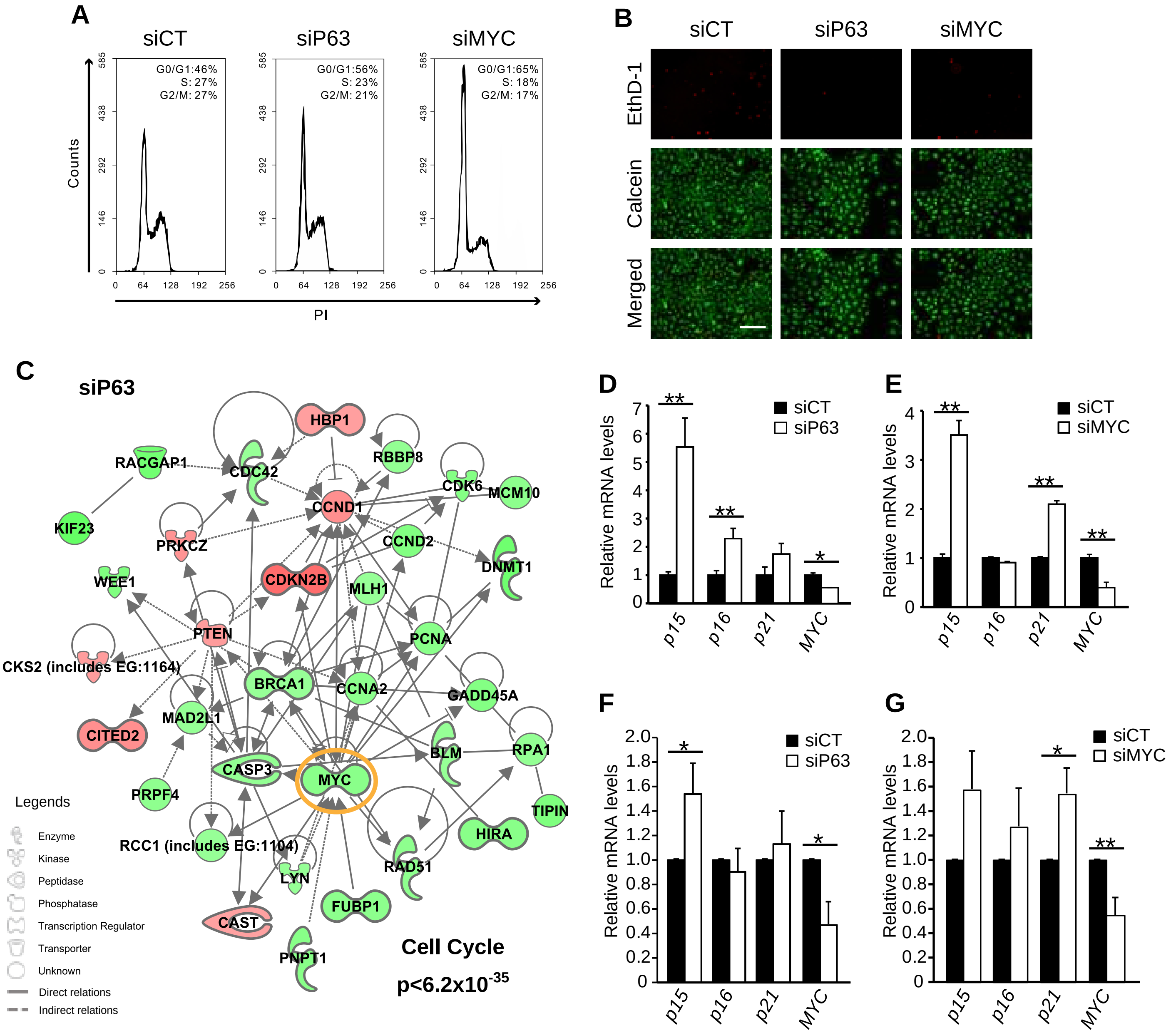
A
siCT
siP63
siMYC
G0/G1:46%
S: 27%
G2/M: 27%
G0/G1:56%
S: 23%
G2/M: 21%
G0/G1:65%
S: 18%
G2/M: 17%
Counts
PI
B
siCT
siP63
siMYC
EthD-1
Calcein
Merged
C
siP63
HBP1
RACGAP1
CDC42
RBBP8
CDK6
MCM10
CCND1
KIF23
PRKCZ
CCND2
DNMT1
WEE1
CDKN2B
MLH1
PTEN
PCNA
CKS2 (includes EG:1164)
BRCA1
CCNA2
GADD45A
MAD2L1
CITED2
BLM
RPA1
CASP3
MYC
PRPF4
TIPIN
RCC1 (includes EG:1104)
HIRA
RAD51
LYN
FUBP1
CAST
PNPT1
Cell Cycle
p<6.2x10-35
Legends
Enzyme
Kinase
Peptidase
Phosphatase
Transcription Regulator
Transporter
Unknown
Direct relations
Indirect relations
D
siCT
siP63
Relative mRNA levels
p15
p16
p21
MYC
E
siCT
siMYC
Relative mRNA levels
p15
p16
p21
MYC
F
siCT
siP63
Relative mRNA levels
p15
p16
p21
MYC
G
siCT
siMYC
Relative mRNA levels
p15
p16
p21
MYC

Figure 3

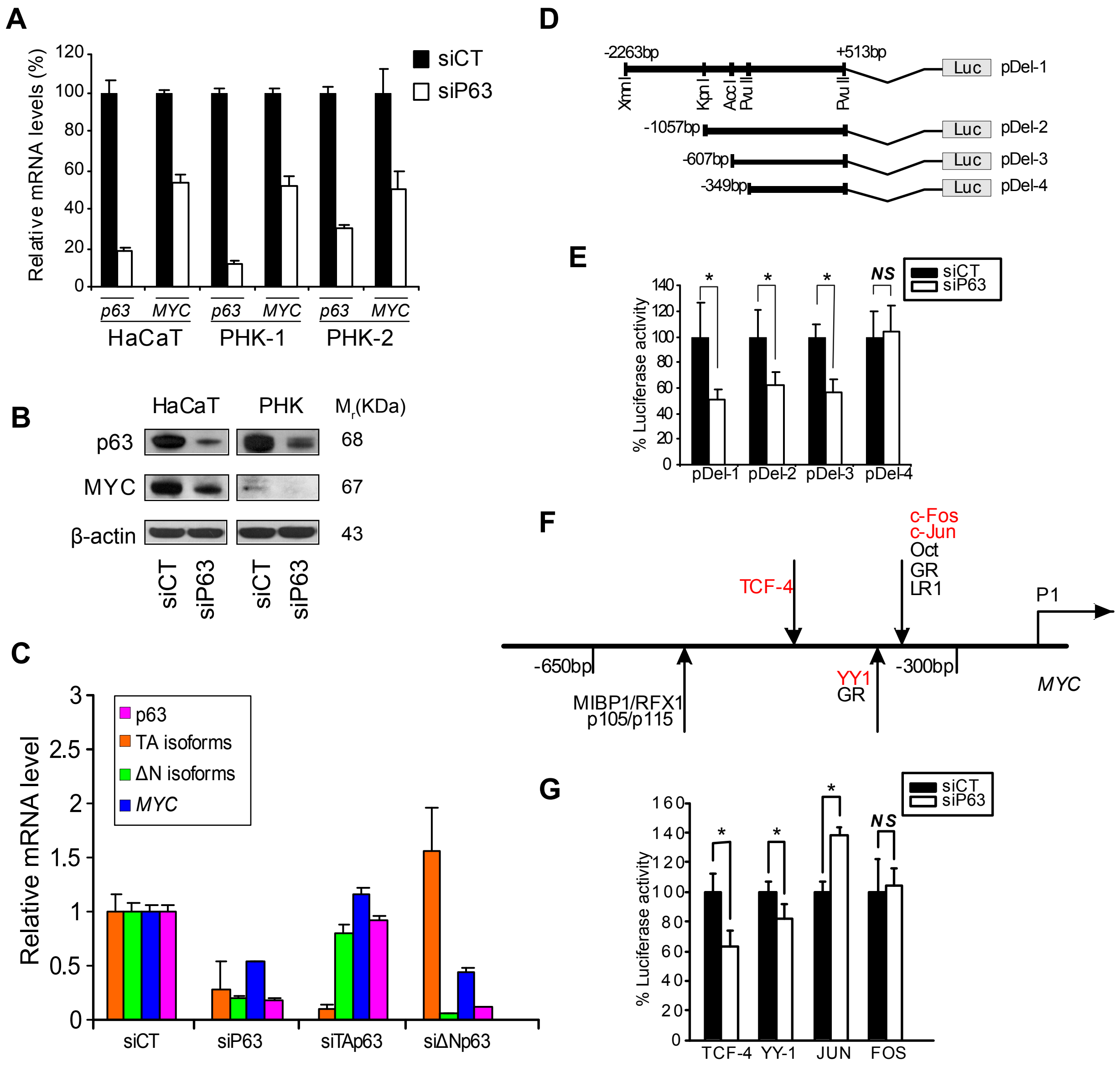
A
Relative mRNA levels (%)
siCT
siP63
p63
MYC
HaCaT
PHK-1
PHK-2
B
HaCaT
PHK
Mr(KDa)
p63
68
MYC
67
β-actin
43
siCT
siP63
C
Relative mRNA level
p63
TA isoforms
ΔN isoforms
MYC
siCT
siP63
siTAp63
siΔNp63
D
-2263bp
+513bp
Xmn I
Kpn I
Acc I
Pvu II
Luc
pDel-1
-1057bp
pDel-2
-607bp
pDel-3
-349bp
pDel-4
E
% Luciferase activity
NS
F
c-Fos
c-Jun
Oct
GR
LR1
TCF-4
P1
-650bp
-300bp
MYC
YY1
GR
MIBP1/RFX1
p105/p115
G
TCF-4
YY-1
JUN
FOS

Figure 4

A

Pathways	Genes	Effects in Pathways	Microarray siP63
Notch	JAG1	activator	↓
	DLL1	activator	↓
β-catenin /TCF-4	DKK1	inhibitor	↑
	IGFBP6	inhibitor	↑
	KLF6	inhibitor	↑
	HBP-1	inhibitor	↑
	PTEN	inhibitor	↑
	UPA	activator	↓
	MMP13	activator	↓

B

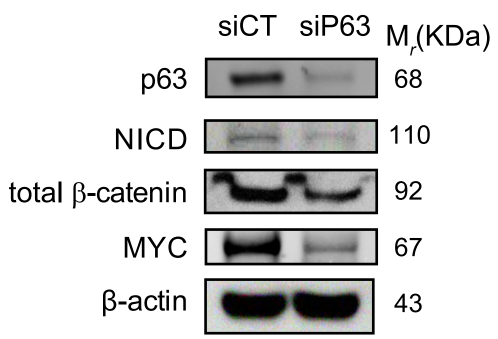

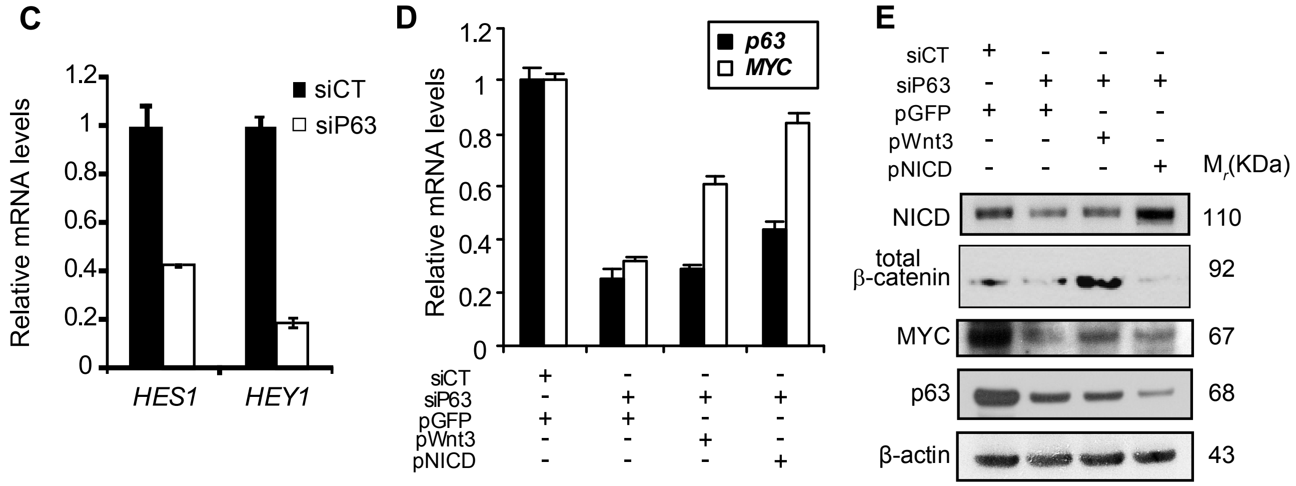

Figure 5

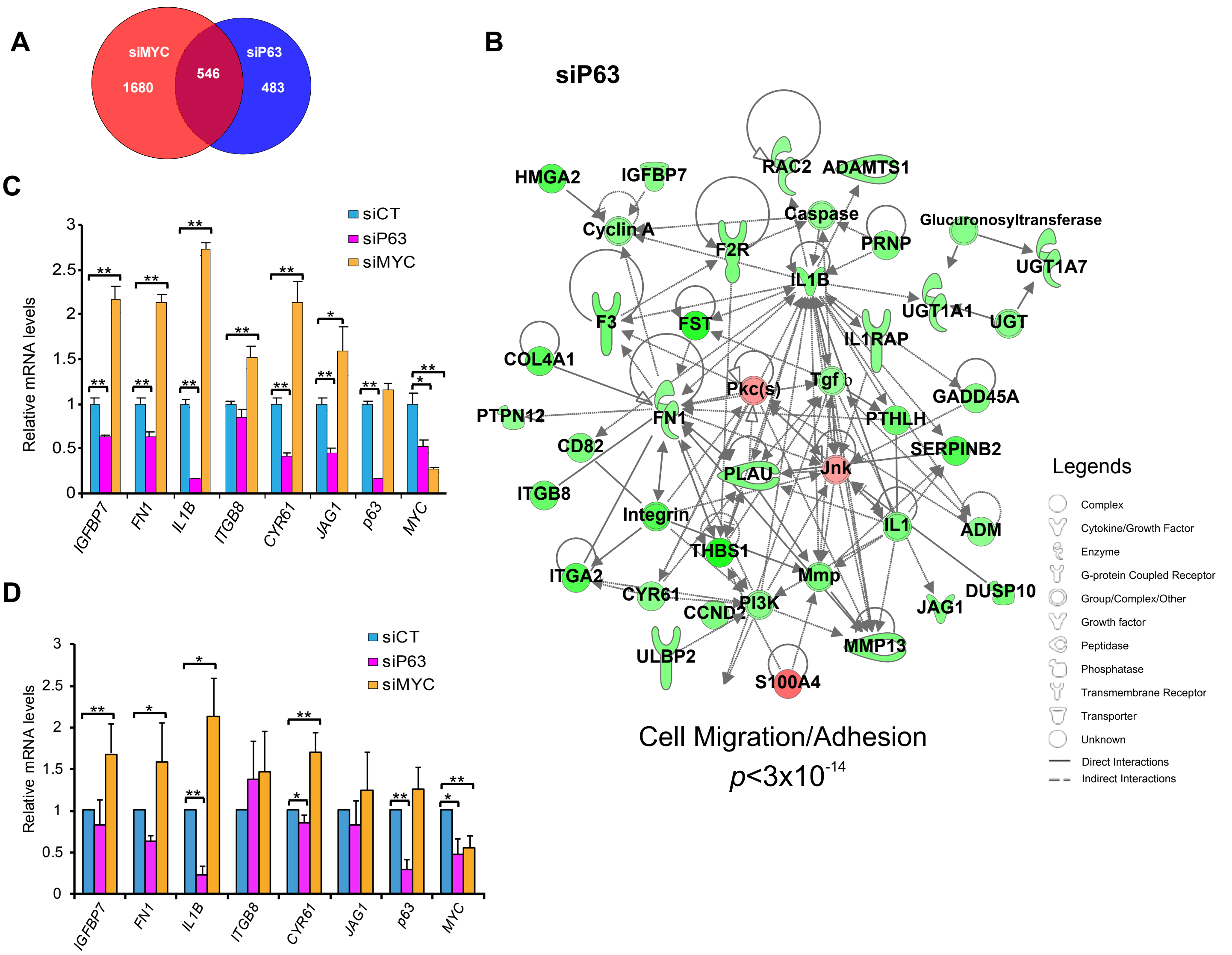
A
siMYC
siP63
1680
546
483
B
siP63
HMGA2
IGFBP7
RAC2
ADAMTS1
Cyclin A
Caspase
Glucuronosyltransferase
F2R
PRNP
UGT1A7
IL1B
UGT1A1
UGT
F3
FST
IL1RAP
COL4A1
Pkc(s)
Tgf b
GADD45A
PTPN12
FN1
PTHLH
CD82
SERPINB2
PLAU
Jnk
ITGB8
Integrin
IL1
ADM
THBS1
ITGA2
Mmp
DUSP10
CYR61
PI3K
JAG1
CCND2
ULBP2
MMP13
S100A4
Cell Migration/Adhesion
p<3x10-14
Legends
Complex
Cytokine/Growth Factor
Enzyme
G-protein Coupled Receptor
Group/Complex/Other
Growth factor
Peptidase
Phosphatase
Transmembrane Receptor
Transporter
Unknown
Direct Interactions
Indirect Interactions
C
siCT
siP63
siMYC
Relative mRNA levels
IGFBP7
FN1
IL1B
ITGB8
CYR61
JAG1
p63
MYC
D
siCT
siP63
siMYC
Relative mRNA levels
IGFBP7
FN1
IL1B
ITGB8
CYR61
JAG1
p63
MYC

Figure 6

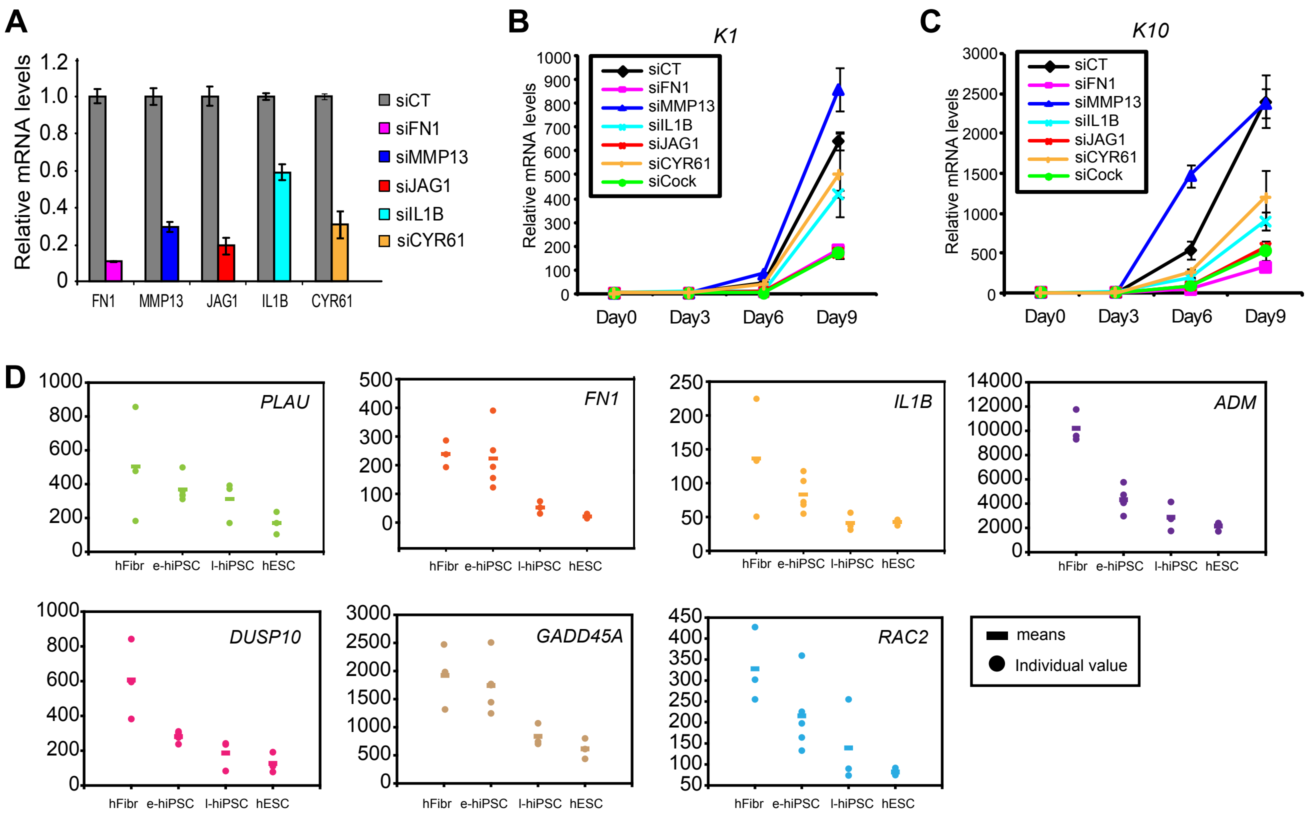
A
Relative mRNA levels
1.2
1.0
0.8
0.6
0.4
0.2
0
FN1
MMP13
JAG1
IL1B
CYR61
siCT
siFN1
siMMP13
siJAG1
siIL1B
siCYR61
B
K1
Relative mRNA levels
siCT
siFN1
siMMP13
siIL1B
siJAG1
siCYR61
siCock
Day0
Day3
Day6
Day9
C
K10
Relative mRNA levels
siCT
siFN1
siMMP13
siIL1B
siJAG1
siCYR61
siCock
Day0
Day3
Day6
Day9
D
PLAU
FN1
IL1B
ADM
DUSP10
GADD45A
RAC2
hFibr
e-hiPSC
l-hiPSC
hESC
means
Individual value

Figure 7

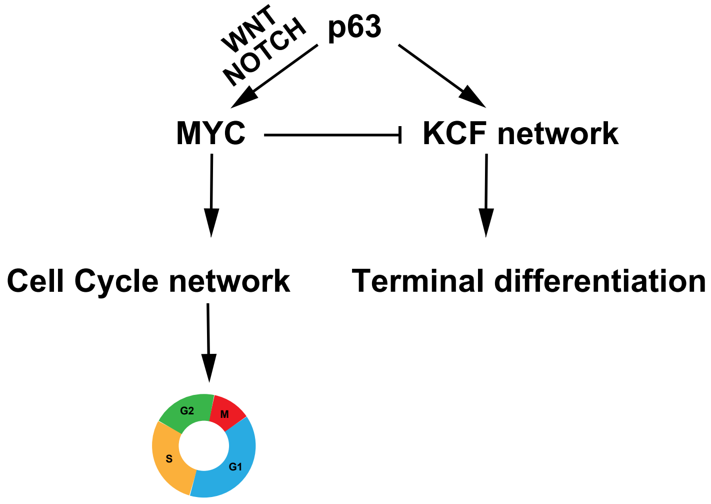
WNT
NOTCH
p63
MYC
KCF network
Cell Cycle network
Terminal differentiation
G2
M
S
G1

Table 1

Gene ID	GenBank Accession Number	Phenotype
**CCND2**	NM_001759	abnormal external granule cell layer morphology
COL17A1	NM_130778	abnormal epidermal layer morphology;skin lesions
DFNA5	NM_004403	decreased cochlear outer hair cell number
DST	NM_001723	abnormal skin pigmentation
**F2R**	NM_001992	abnormal skin condition
**F3**	NM_001993	abnormal skin condition
**FN1**	NM_002026	skin lesions
**FST**	NM_006350	thickened epidermis
**GADD45A**	NM_001924	increased sensitivity to skin irradiation
**HMGA2**	NM_003483	long hair
**IL1RAP**	NM_002182	abnormal skin condition/ morphology
**ITGA2**	NM_002203	abnormal skin condition
**JAG1**	NM_000214	abnormal cochlear hair cell morphology
**Jnk**		decreased hair follicle number
KRT6A	NM_005554	abnormal coat/ hair morphology
**PI3K**		skin lesions
**PTHLH**	NM_002820	thin epidermis
**THBS1**	NM_003246	abnormal skin condition
**PRNP**	NM_000311	abnormal skin condition

Supplemental Information

Figure S1. Time-course analysis of p63 or MYC expression after siP63 or siMYC transfection. (**A, B**) p63 expression levels were quantified using qRT-PCR (**A**) and western blots (**B**) over the course of 10 days. (**C**) MYC expression levels in p63-knockdown cells were quantified using qRT-PCR. (**D**) MYC expression levels in siMYC were quantified by qRT-PCR and western blots (**E**) over the course of 10 days. Samples were extracted every 2 days from day 2 to day 10.

Figure S2. MYC expression in p63-depleted keratinocytes in mRNA level (**A**) and protein level (**B**). BR means biological replicate.

Figure S3. Genetic networks regulating cell cycle (**A**) and cell migration and adhesion (**B**) were generated by Ingenuity Pathway Analysis (IPA) for siMYC. Same networks as identified in siP63.

Figure S4. Data mining of MYC expression in GEO. Other MYC expression data were found in GEO, with p63 knockdown in several epithelial cell lines.

Figure S5. Ectopic expression of TAp63γ or ΔNp63α was not able to upregulate MYC expression. HaCaT cells were transfected with plasmids containing the ORF of TAp63γ, ΔNp63α, or GFP (control). 48 hours post-transfection, RNA was extracted and measured by qRT-PCR.

Figure S6. Gene Ontology (GO) analysis for the 546 common genes in Fig. 5a. (**A-D**) GO terms were significantly enriched in downregulated genes in the green columns (**A**, siMYC; **B**, siP63) or in upregulated genes in the red columns (**C**, siMYC; **D**, siP63). Significantly enriched GO

terms are represented in a histogram as the –log ($p<0.05$, Fisher's exact test). The numbers indicated above columns represent the number of genes classified into each category. The gray dashed lines represent the significance threshold.

Table S1. List of all PCR primers used in this study.

Table S2. List of genes differentially expressed in siMYC or siP63 transcriptome analysis.

Table S3. List of common genes modulated inversely in the siMYC and siP63 transcriptomes.

Figure S1

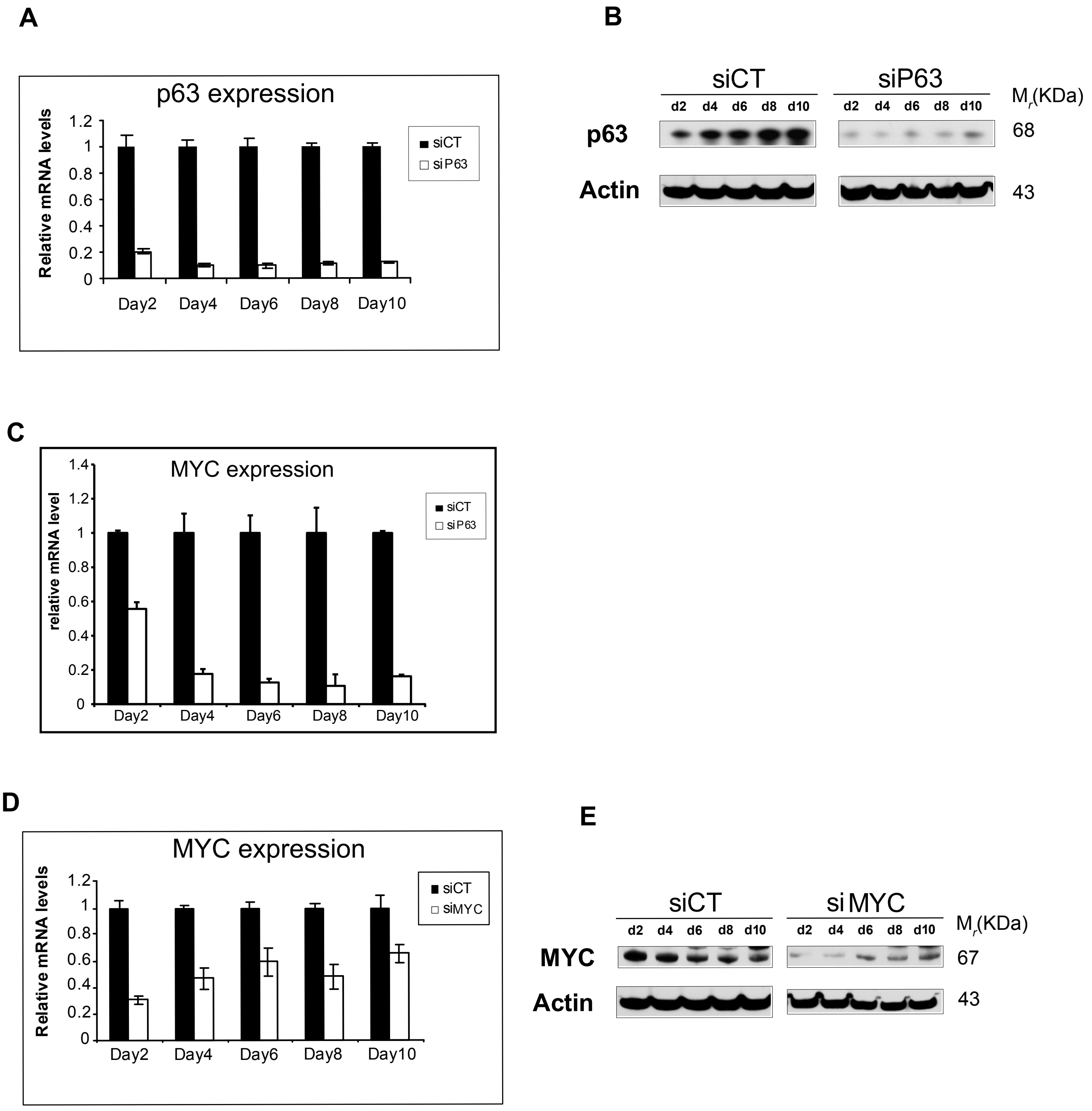
A
p63 expression
Relative mRNA levels
1.2
1
0.8
0.6
0.4
0.2
0
Day2
Day4
Day6
Day8
Day10
siCT
si P63
B
siCT
siP63
d2 d4 d6 d8 d10
d2 d4 d6 d8 d10
M_r(KDa)
p63
68
Actin
43
C
MYC expression
relative mRNA level
1.4
1.2
1
0.8
0.6
0.4
0.2
0
Day2
Day4
Day6
Day8
Day10
siCT
si P63
D
MYC expression
Relative mRNA levels
1.2
1
0.8
0.6
0.4
0.2
0
Day2
Day4
Day6
Day8
Day10
siCT
siMYC
E
siCT
si MYC
d2 d4 d6 d8 d10
d2 d4 d6 d8 d10
M_r(KDa)
MYC
67
Actin
43

Figure S2

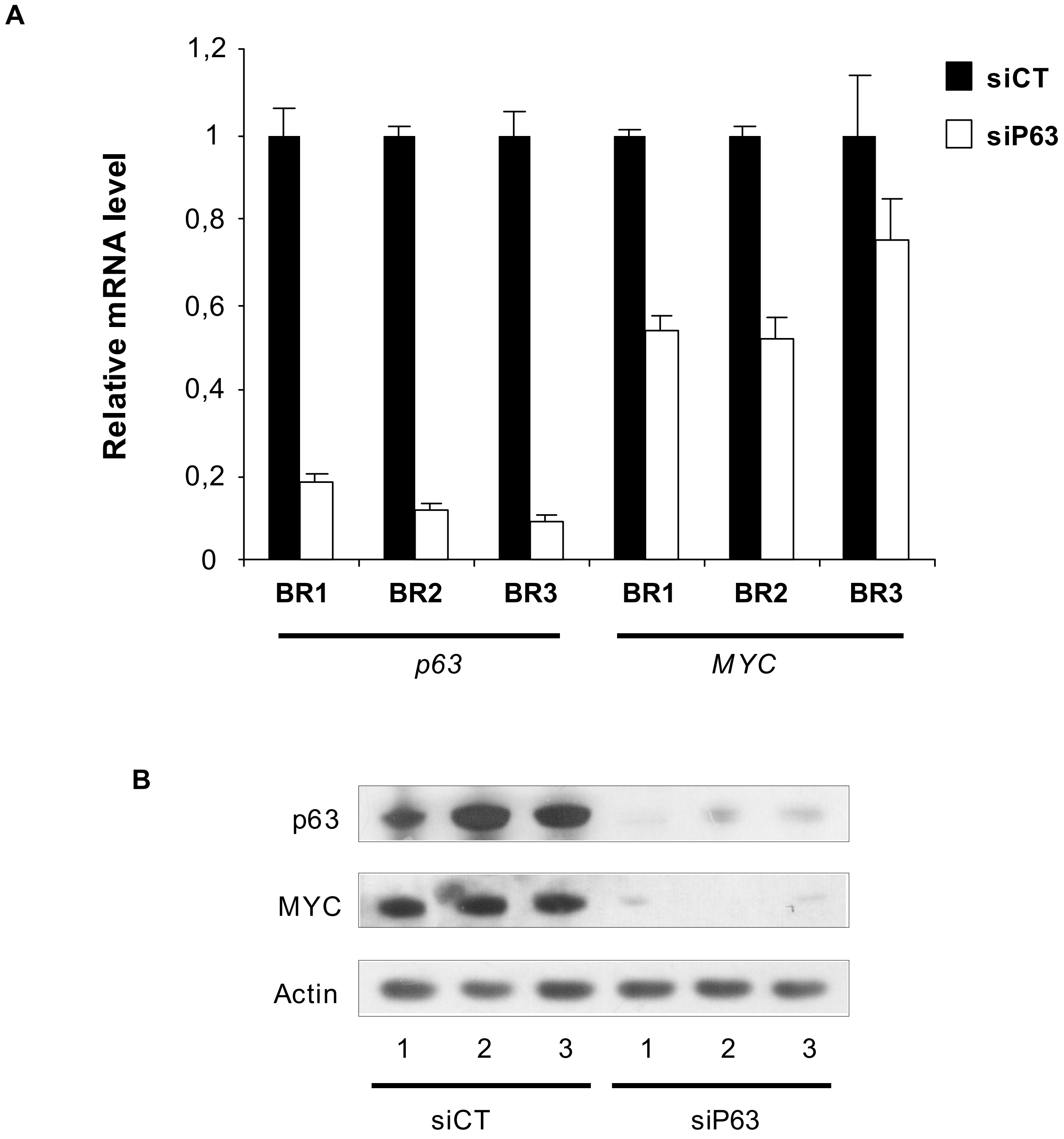
A
1,2
1
0,8
0,6
0,4
0,2
0
Relative mRNA level
siCT
siP63
BR1
BR2
BR3
BR1
BR2
BR3
p63
MYC
B
p63
MYC
Actin
1
2
3
1
2
3
siCT
siP63

Figure S3

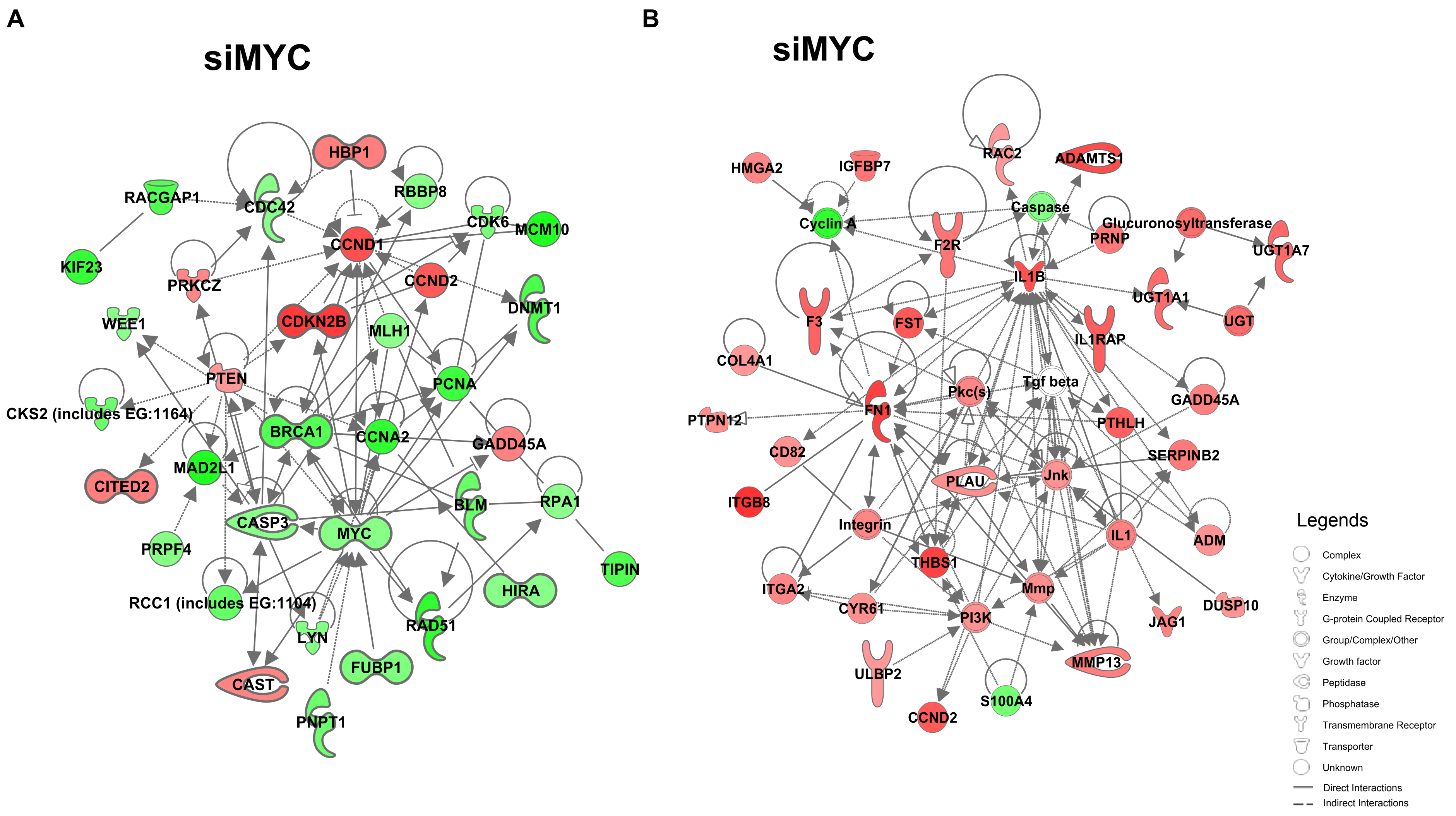

Cell Cycle

Cell migration/adhesion

Figure S4

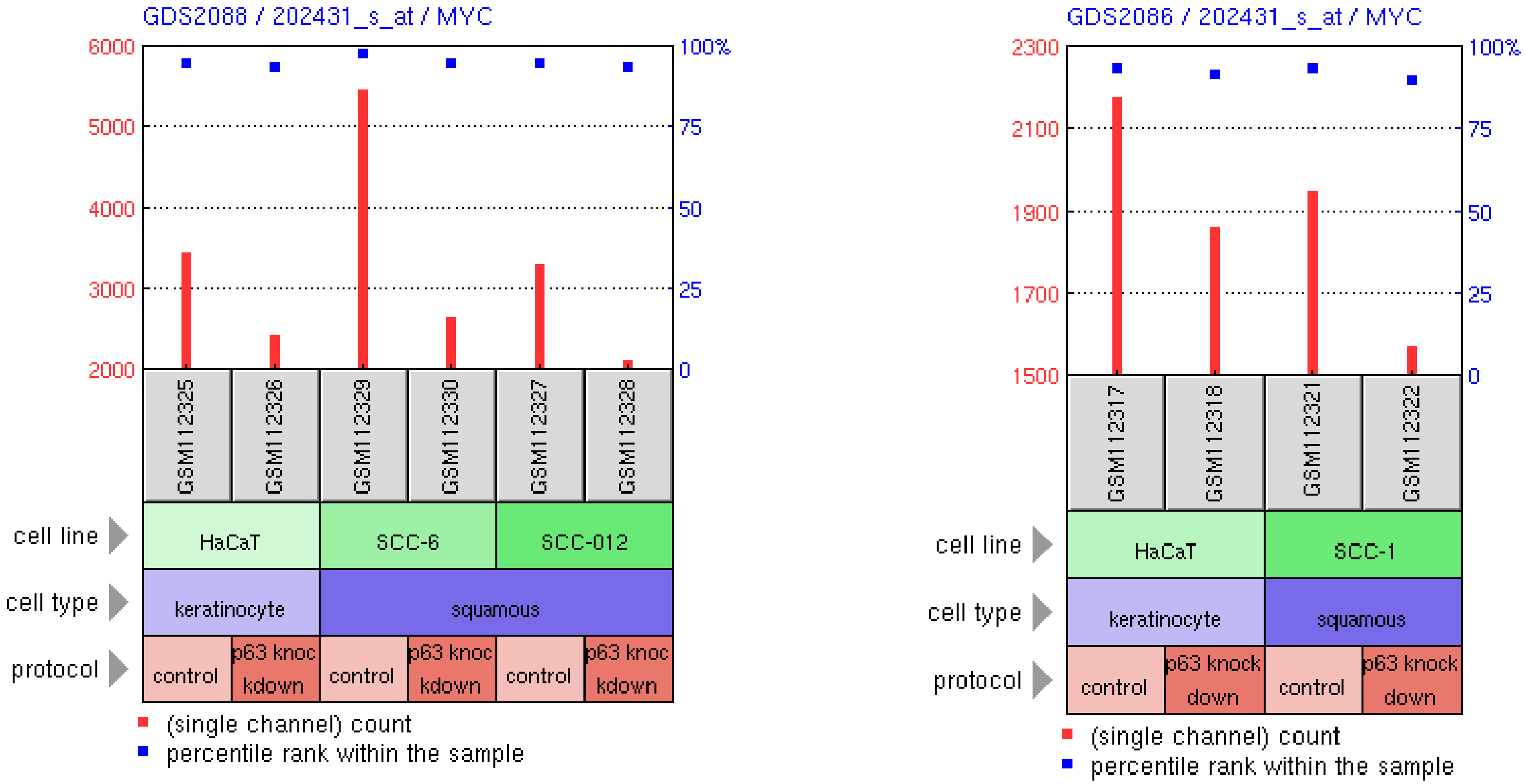

GSM112325: HaCaT cells transfected with control vector

GSM112326: HaCaT cells transfected with p63 siRNA

GSM112329: SCC-6 cells transfected with control vector

GSM112330: SCC-6 cells transfected with p63 siRNA

GSM112327: SCC-012 cells transfected with control vector

GSM112328: SCC-012 cells transfected with p63 siRNA

GSM112317: HaCaT RG cells transfected with control vector 1

GSM112318: HaCaT RG cells transfected with p63 siRNA 1

GSM112321: SCC-1 cells transfected with control vector 1

GSM112322: SCC-1 cells transfected with p63 siRNA 1

Figure S5

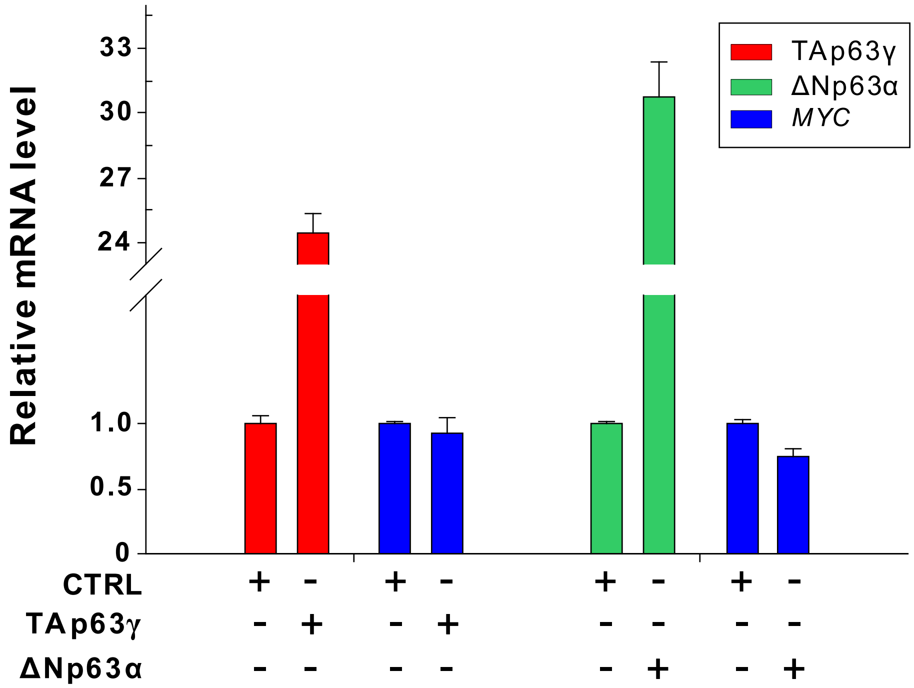
Relative mRNA level
33
30
27
24
1.0
0.5
0
TAp63γ
ΔNp63α
MYC
CTRL
TAp63γ
ΔNp63α
+ - + - + - + -
- + - + - - - -
- - - - - + - +

Figure S6

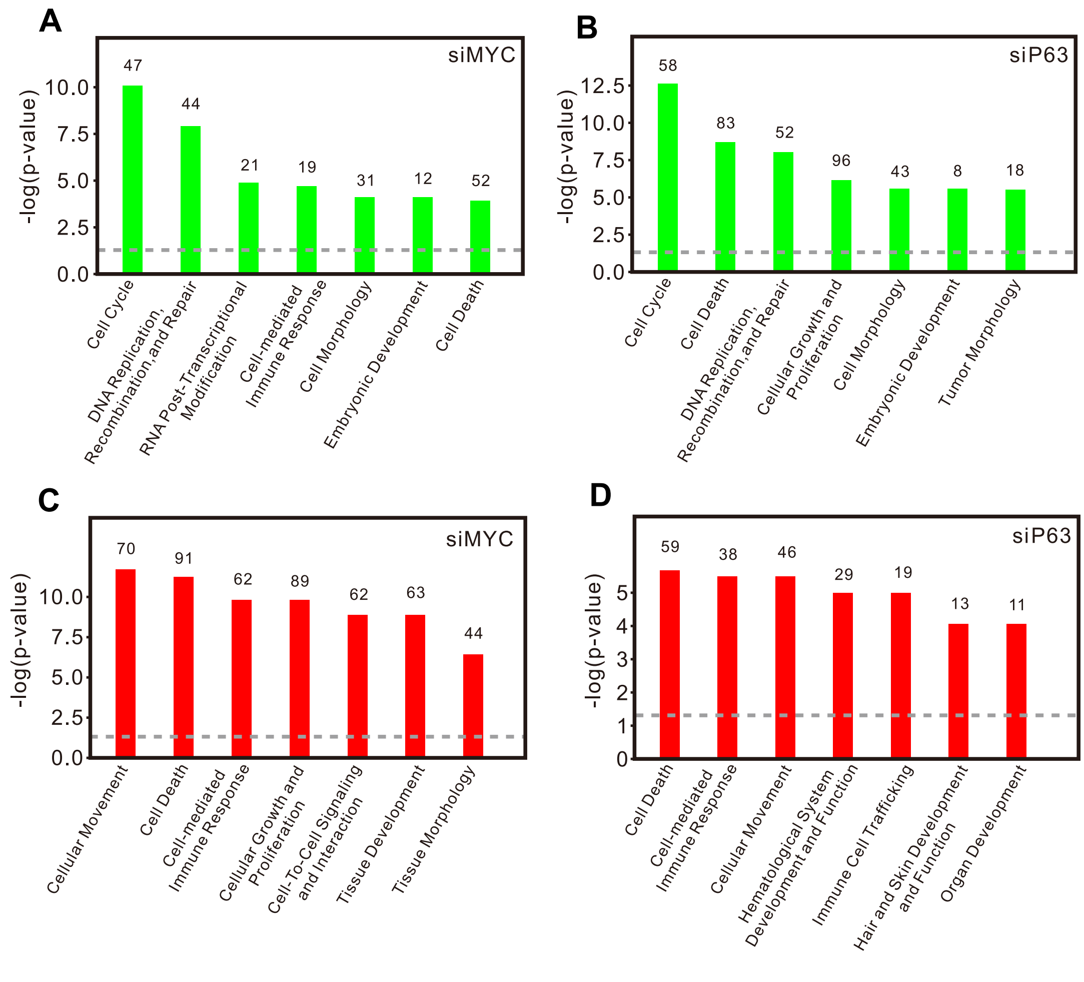
A
siMYC
-log(p-value)
10.0
7.5
5.0
2.5
0.0
47
44
21
19
31
12
52
Cell Cycle
DNA Replication, Recombination, and Repair
RNA Post-Transcriptional Modification
Cell-mediated Immune Response
Cell Morphology
Embryonic Development
Cell Death
B
siP63
-log(p-value)
12.5
10.0
7.5
5.0
2.5
0.0
58
83
52
96
43
8
18
Cell Cycle
Cell Death
DNA Replication, Recombination, and Repair
Cellular Growth and Proliferation
Cell Morphology
Embryonic Development
Tumor Morphology
C
siMYC
-log(p-value)
10.0
7.5
5.0
2.5
0.0
70
91
62
89
62
63
44
Cellular Movement
Cell Death
Cell-mediated Immune Response
Cellular Growth and Proliferation
Cell-To-Cell Signaling and Interaction
Tissue Development
Tissue Morphology
D
siP63
-log(p-value)
5
4
3
2
1
0
59
38
46
29
19
13
11
Cell Death
Cell-mediated Immune Response
Cellular Movement
Hematological System Development and Function
Immune Cell Trafficking
Hair and Skin Development and Function
Organ Development

Table S3

List of common genes modulated oppositely in siTP63 and siMYC transcriptome. Fold changes were given.

**Gene Name**	**GeneBank**	**si*MYC***	**si*P63***
ACAT2	NM_005891	-1,20	1,25
ADAMTS1	NM_006988	2,13	-1,38
ADM	NM_001124	1,29	-1,31
AKR1C2	NM_001354	1,26	-1,26
AMIGO2	NM_181847	1,77	-1,37
ANXA8L2	NM_001630	1,49	-1,61
C20ORF39	NM_024893	1,59	-1,27
CCND2	NM_001759	1,96	-1,46
CD82	NM_002231	1,30	-1,52
CKS2	NM_001827	-1,70	1,26
COL12A1	NM_004370	1,29	-1,47
COL17A1	NM_000494	1,56	-1,31
COL4A1	NM_001845	1,22	-1,88
CSTA	NM_005213	1,33	-1,80
CYR61	NM_001554	1,30	-1,33
DCXR	NM_016286	-1,39	1,24
DDX10	AB001343	-1,36	-1,25
DFNA5	NM_004403	2,03	-1,21
DKK3	NM_013253	1,98	-2,13
DST	NM_001723	1,37	-2,04
DUSP10	NM_007207	1,27	-1,59
DUSP6	NM_001946	2,17	-1,47
F2R	NM_001992	1,65	-1,49
F3	NM_001993	1,86	-1,69
FAM3C	NM_014888	1,44	-1,51
FLJ10357	NM_018071	1,34	-1,24
FN1	NM_002026	2,27	-1,32
FST	NM_006350	1,98	-2,46
GADD45A	NM_001924	1,49	-1,43
GBP3	NM_018284	1,72	-1,21
GOLT1B	NM_016072	1,31	-1,21
GPX4	NM_002085	-1,31	1,25
HES7	NM_032580	-1,33	1,27
HLA-B	NM_005514	1,47	-1,25
HMGA2	NM_003483	1,43	-1,98
IGFBP7	NM_001553	1,65	-1,36
IL1B	NM_000576	2,07	-1,52
IL1RAP	NM_002182	1,92	-1,36
ITGA2	NM_002203	1,44	-2,01
ITGB8	BC042028	2,60	-1,75
JAG1	NM_000214	1,47	-1,42
KIAA1128	NM_018999	1,25	-1,23
KRT6A	NM_005554	1,28	-1,46
MMP13	NM_002427	1,52	-1,55
NMU	NM_006681	-1,34	1,41
NUAK1	NM_014840	1,44	-1,32
PAIP2	NM_016480	1,26	-1,49
PLAU	NM_002658	1,33	-1,52
PLK2	AK098163	1,48	-1,26
PRNP	NM_000311	1,59	-1,46
PTHLH	NM_002820	1,88	-1,68
PTPN12	NM_002835	1,35	-1,27
PTPRU	NM_005704	1,27	-1,38

RAC2	NM_002872	1,22	-1,24
RGS20	NM_003702	1,28	-1,27
S100A4	NM_002961	-1,58	1,92
SERPINB2	NM_002575	1,58	-2,01
SERPINB7	NM_003784	1,52	-1,52
SORL1	AA424516	1,43	-1,59
THBS1	NM_003246	2,24	-2,60
TXNRD1	NM_003330	1,22	-1,62
UGT1A1	NM_000463	1,75	-1,32
UGT1A7	NM_019077	1,78	-1,38
ULBP2	NM_025217	1,21	-1,44
VAMP3	NM_004781	1,29	-1,23
ZFAND6	NM_019006	-1,59	1,27
